# Chromosome-level genome assembly of the aquatic plant *Nymphoides indica* reveals transposable element bursts and NBS-LRR gene family expansion shedding light on its invasiveness

**DOI:** 10.1093/dnares/dsac022

**Published:** 2022-06-25

**Authors:** Jing-Shan Yang, Zhi-Hao Qian, Tao Shi, Zhi-Zhong Li, Jin-Ming Chen

**Affiliations:** Key Laboratory of Aquatic Botany and Watershed Ecology, Wuhan Botanical Garden, Chinese Academy of Sciences, Wuhan 430074, China; Center of Conservation Biology, Core Botanical Gardens, Chinese Academy of Sciences, Wuhan 430074, China; University of Chinese Academy of Sciences, Beijing 100049, China; Key Laboratory of Aquatic Botany and Watershed Ecology, Wuhan Botanical Garden, Chinese Academy of Sciences, Wuhan 430074, China; Center of Conservation Biology, Core Botanical Gardens, Chinese Academy of Sciences, Wuhan 430074, China; University of Chinese Academy of Sciences, Beijing 100049, China; Key Laboratory of Aquatic Botany and Watershed Ecology, Wuhan Botanical Garden, Chinese Academy of Sciences, Wuhan 430074, China; Center of Conservation Biology, Core Botanical Gardens, Chinese Academy of Sciences, Wuhan 430074, China; Key Laboratory of Aquatic Botany and Watershed Ecology, Wuhan Botanical Garden, Chinese Academy of Sciences, Wuhan 430074, China; Center of Conservation Biology, Core Botanical Gardens, Chinese Academy of Sciences, Wuhan 430074, China; Key Laboratory of Aquatic Botany and Watershed Ecology, Wuhan Botanical Garden, Chinese Academy of Sciences, Wuhan 430074, China; Center of Conservation Biology, Core Botanical Gardens, Chinese Academy of Sciences, Wuhan 430074, China

**Keywords:** *Nymphoides indica*, invasive, chromosome-level genome, transposable elements, NBS-LRR gene family

## Abstract

*Nymphoides indica*, an aquatic plant, is an invasive species that causes both ecological and economic damage in North America and elsewhere. However, the lack of genomic data of *N. indica* limits the in-depth analysis of this invasive species. Here, we report a chromosome-level genome assembly of nine pseudochromosomes of *N. indica* with a total size of ∼ 520 Mb. More than half of the *N. indica* genome consists of transposable elements (TEs), and a higher density of TEs around genes may play a significant role in response to an ever-changing environment by regulating the nearby gene. Additionally, our analysis revealed that *N. indica* only experienced a gamma (γ) whole-genome triplication event. Functional enrichment of the *N. indica*-specific and expanded gene families highlighted genes involved in the responses to hypoxia and plant–pathogen interactions, which may strengthen the ability to adapt to external challenges and improve ecological fitness. Furthermore, we identified 160 members of the nucleotide-binding site and leucine-rich repeat gene family, which may be linked to the defence response. Collectively, the high-quality *N. indica* genome reported here opens a novel avenue to understand the evolution and rapid invasion of *Nymphoides* spp.

## 1. Introduction

*Nymphoides indica* (L.) Kuntze (Menyanthaceae) is a perennial floating-leaved aquatic plant (water snowflake) and widely distributed in pantropical regions, such as tropical America, Asia, and Australia.^1–3^ It is a heterostylous self-incompatible species that can reproduce sexually by seeds, asexually by producing a type of turion in the roots that can easily separate from the parent plant, or by adventitious plantlet growth.^4^^,^^5^ The plant grows mainly in lakes, ponds, rice fields, and slow-moving rivers. Due to human activities, the local populations of *N. indica* in Japan have been greatly reduced and it is now listed as an endangered species in the country.^5^ However, it has been intentionally introduced in many countries or regions as an ornamental plant in waters where it escapes into the wild and rapidly expands.^6^ In Florida, Texas, and North Carolina in the USA, *N. indica* has been found in numerous water bodies and listed as a noxious weed.^6–8^

Similar to *N*. *indica*, several other species in the genus *Nymphoides*, such as *N*. *cristata* (crested floating heart) and *N*. *peltata* (yellow floating heart), also have wide distribution ranges and are regarded as noxious weeds in some regions and countries.^9–11^ Owing to their well-defined vegetative propagation capability,^10^ these species can rapidly spread into various waters and form dense floating canopies covering the water surface. This can significantly reduce light penetration into water and the dissolved oxygen in water, causing serious damage to aquatic ecosystems and further reducing native standing plant biomass and biodiversity.^12^^,^^13^ However, little is known about the mechanisms underlying this rapid invasion.

Studying the genetic basis of weeds/invasive species enables the identification of the factors responsible for their successful range expansion. Whole-genome sequencing offers opportunities to link the environment with innovation. For instance, by analysing the ancient whole-genome duplications (WGDs) of flowering plants, many studies show that WGDs are common and coincide with several paleoclimate change events.^14^^,^^15^ The expansion of genetic material from the genomic duplication may result in key innovations and phenotypic novelty, which could promote adaptation and enhance survival.^16–18^ By studying transposable elements (TEs) at the whole genomic scale, researchers found that TEs may influence regulatory networks and provide a mechanism for genome evolution and adaptation.^19^^,^^20^ In addition, several population genomic studies have confirmed that in the process of plant invasions, TEs can produce genetic variation and generate rapid phenotypic variations to facilitate adaptation.^21^^,^^22^ By investigating the expansions and contractions of gene families in plant genomes, such as the nucleotide-binding site and leucine-rich repeat (NBS-LRR) gene family, which includes the majority of disease-resistance genes in plants, previous studies have found that the rapid expansion and/or contraction of the NBS-LRR gene family plays a significant role in quick adaptation to challenges from novel pathogens.^23^^,^^24^ To date, only a few weedy/invasive plant species genomes have been sequenced (e.g. *Conyza canadensis*;^25^*Mikania micrantha*;^26^*Microstegium vimineum*^;27^ and *Phragmites australis*^28^) no genome has been reported for the family Menyanthaceae (Asterales). *Our understanding* of the adaptations of the weeds or widely distributed plant species is still limited, especially for aquatic plants that include a large number of globally notorious invasive species.

In this study, we present a high-quality chromosome-level genome assembly of *N. indica* using a combination of PacBio sequencing, Illumina sequencing, and HiC technology. Comparative genomic analyses were conducted to investigate WGDs and gene family expansions and contractions, with an emphasis on the NBS-LRR gene family. In addition, we explored the effect of TEs on the rapid invasion of *N*. *indica*. The results provide vital insights into the quick dispersal of aquatic plants to diverse aquatic environments and their potential invasiveness.

## 2. Materials and methods

### 2.1. Genome sequencing

Wild *N. indica* samples used for sequencing were collected from Haikou, Hainan Province, China and planted in a greenhouse at the Wuhan Botanical Garden. The leaves of *N. indica* (2*n* = 18) were collected for *de novo* genome sequencing.^29^ High-quality genomic DNA was extracted from fresh leaves using a modified CTAB method and sequenced on the Illumina HiSeq X Ten (Illumina Inc., San Diego, CA, USA) and PacBio Sequel (Pacific Biosciences of California, Menlo Park, CA, USA) platforms.

RNA data were extracted separately from the four tissues (root, stem, leaf, and flower) using the NEBNext^®^ Ultra RNA Library Prep Kit for Illumina^®^ (NEB, USA). According to the manufacturer’s instructions, each RNA-seq library was purified on a cBot Cluster Generation System using TruSeq PE Cluster Kit v3-cBot-HS (Illumina), and PE150 sequencing was conducted on an Illumina NovaSeq 6000 platform. Quality control of primitive RNA-seq reads was performed using FastQC v0.11.7 (http://www.bioinformatics.babraham.ac.uk/projects/fastqc/, 10 January 2022, date last accessed), and poor-quality reads were trimmed using Trimmomatic v0.36.^30^

Briefly, the Hi-C libraries of *N. indica* were constructed using the following steps: (i) fresh leaves were cut and fixed in formaldehyde for crosslinking, chromatin digestion with the four-cutter restriction enzyme *Mbo*I, (ii) DNA ligation with the T4 DNA ligation enzyme, and (iii) purification and fragmentation (the purified DNA was sheared to a length of ∼ 400 bp). Finally, the Hi-C libraries were sequenced on the Illumina HiSeq X Ten platform (San Diego, CA, USA) in PE150 mode.

### 2.2. Genome size evaluation and genome assembly

We estimated the genome size of *N. indica* using the *k-mer* frequency analysis. Illumina clean reads were used to calculate *k*-*mer* distribution by Jellyfish v2.2.3^31^ and then to estimate the genome size and the level of heterozygosity of the genome using GCE v1.0.2.^32^

For genome assembly, quality-controlled PacBio long reads were corrected using the NextCorrect module of NextDenovo v2.1 (https://github.com/Nextomics/NextDenovo, 10 January 2022, date last accessed) with default parameters. Corrected reads were trimmed and assembled using Canu software v1.8^33^ with the Corrected-Error-Rate parameter set at 0.035. Subsequently, the draft assembly was polished using NextPolish v1.3.1.^34^ All PacBio long reads and Illumina short reads were used in all three genome editing iterations for genome polishing. We then performed Purge Haplotigs v1.1.1^35^ to remove redundant sequences and obtain the final contig-level assembly.

In this study, we used Hi-C reads to construct chromosome-level assemblies. The original Hi-C sequenced data were filtered using Hic-Pro v2.11.1.^36^ Then, the clean data were compared with the genome using Juicer v1.6.^37^ Contig ordering, orientation, and chimera splitting was conducted using the 3D-DNA pipeline^38^ under default parameters. Contig misassemblies and scaffold misjoins were manually detected and corrected based on interaction densities from visualization in Juicebox.^39^ Finally, we evaluated the integrity and accuracy of the genome assembly using Benchmarking Universal Single-Copy Orthologs (BUSCO; v4.0.1)^40^ in the Embryophyta_odb10 database.

### 2.3. Genome annotation

We performed RepeatMasker v4.1.0 (http://www.repeatmasker.org, 10 January 2022, date last accessed) to identify and classify the repetitive elements in the *N. indica* genome using the *de novo* repeat library and the Repbase database. A *de novo* repeat library for *N. indica* was built using RepeatModeler v2.0.1 (http://www.repeatmasker.org, 10 January 2022, date last accessed) and the Repbase^41^ database was downloaded from http://www.girinst.org/repbase/) (10 January 2022, date last accessed. Three different procedures were used to predict protein-coding genes in the *N. indica* genome assembly: (i) AUGUSTUS v3.3.3^42^ was used to perform *de novo* prediction; (ii) for homology prediction, the homologous sequences of three Asteraceae species, *Carthamus tinctorius, Chrysanthemum nankingense*, and *Cynara cardunculus* were aligned against the *N. indica* genome using TBLASTN with an e-value cutoff of 1e−5, and the gene structure was predicted using GeneWise v2.4.1;^43^ (iii) Trinity v2.11.0^44^ was conducted to *de novo* assemble all RNA-seq data for transcriptome-based prediction. The assembled transcripts were applied to PASA v2.4.1^45^ and TransDecoder v5.5.0 (https://github.com/TransDecoder/TransDecoder, 10 January 2022, date last accessed) to identify protein-coding regions. Finally, the gene models identified by each method were integrated using EvidenceModeler v1.1.1^46^ and then updated using PASA v2.4.1.^45^

For the functional annotation of genes, all predicted protein-coding genes were mapped to the Swiss-Prot and NR databases using BLASTP searches with an e-value cutoff of 1e−5 and aligned to the eggNOG database v5.0^47^ using eggNOG-mapper v2.^48^ InterProScan v5.47^49^ was used to identify the functional domains and motifs of the gene set with default parameters. We also used PlantRegMap^50^ to identify transcription factors (TFs) in the *N. indica* genome.

### 2.4. TE identification

We used EDTA v1.9.7,^51^ an automated whole-genome *de novo* TE annotation software, for *de novo* prediction of TEs in *N. indica*. The Perl script ‘solo_finder.pl’ within the LTR_retriever v2.9.0^52^ package was used to identify solo-LTRs. In short, an independent LTR sequence located in regions without a similar LTR sequence (presented at a 300 bp distance) that covered at least 80% of the library LTR entry with an alignment score >300 and length >100 bp were identified as solo LTR. To estimate the removal ratio of long terminal repeat retrotransposons (LTR-RTs), the ratio of solo LTRs to intact LTRs (S/I) was calculated based on the number of solo LTR over the number of intact LTR.

The insertion time of the LTR-RTs was estimated using the formula *T* = K/2r, where the substitution rate of *r* = 7 × 10^−9^ per site per year^53^ was adopted, and the sequence divergence (K) was estimated using the Kimura two-parameter model.

In addition, we compared the distribution of TEs in 100-bp bins in the 2 kb upstream and downstream regions of genes between the invasive species *N. indica*, *C. tinctorius*, *C. cardunculus*, *C. nankingense*, and *M.micrantha*, and non-invasive species *Coffea canephora* and *Vitis vinifera*.

### 2.5. Phylogenomic and gene family evolution analyses

Protein sequences of *N. indica*, *Arabidopsis thaliana*, *Amborella trichopoda*, *C. canephora*, *V. vinifera*, *Helianthusannuus*, *C. cardunculus*, *Oryza sativa*, *Euryale ferox*, *Nymphaea colorata*, *Nelumbo nucifera*, *Zostera marina*, *Spirodela polyrhiza*, and *Tetracentron sinense* were retrieved from public databases (Supplementary Table S1) and used for evolutionary analysis. Orthologous genes among genomes were detected using OrthoFinder v2.7.7^54^ with the default parameters. We used MUSCLE v3.8.31^55^ to align the protein sequences of single-copy orthologous genes and convert the alignments into codon sequences by PAL2NAL v14.1.^56^ After Gblocks v0.91^57^ eliminated non-conservative or divergent regions, we extracted the third codon positions from aligned codon sequences to construct the phylogenetic trees by IQ-TREE v2.1.2.^58^ The divergence time was estimated using the MCMCtree programme of PAML v4.9^59^ based on the following constraints for time calibration: rosids and asterids (110–130 Mya), Asteraceae and non-Asteraceae (>80 Mya),^60^ monocots and dicot (140–150 Mya),^61^*A. trichopoda*, and *N. colorata* (173–199 Mya). The last calibrated time point was obtained from Timetree (http://www.timetree.org/ , 10 January 2022, date last accessed). Cafe v4.2^62^ was used to evaluate the expansion and contraction of the gene families for each lineage. Gene Ontology (GO) and Kyoto Encyclopedia of Genes and Genomes (KEGG) enrichment analyses for gene families of unique, expanded, and contracted genes were performed using the OmicShare tools (https://www.omicshare.com/tools , 10 January 2022, date last accessed).

### 2.6. Whole-genome duplication

We performed a comparison between *N. indica* and two other species (*V. vinifera* and *C. canephora*) to detect WGD events in the *N. indica* genome. Paralogous and orthologous gene pairs were searched via all-versus-all protein sequence comparisons using BLASTP with an e-value cutoff of 1e-5. Intragenomic and intergenomic syntenic blocks were searched for using MCScanX^63^ with default parameters. The Perl script ‘add_ka_and_ks_to_collinearity.pl’ from the MCScanX package was employed to calculate synonymous substitutions per synonymous site (*Ks*) in *N. indica*, *V. vinifera*, and *C. canephora*. The fitting *Ks* distribution was plotted using WGDI v0.4.7.^64^ To further confirm the WGD events, synteny patterns were analysed between *N. indica*, *V. vinifera*, and *C. canephora* based on the intergenomic synteny regions using MCscan (Python-version, https://github.com/tanghaibao/jcvi/wiki/MCscan , 10 January 2022, date last accessed).

### 2.7. Analysis of NBS-LRR genes

All nucleotide-binding site leucine-rich repeat (NBS-LRR) protein sequences of *A. thaliana* were downloaded from RefPlantNLR^65^ and the NBS (NB-ARC) domain (PF00931) was retrieved from the Pfam database (http://pfam.xfam.org/ , 10 January 2022, date last accessed). First, the NBS-LRR protein sequences of *A. thaliana* were used as queries to search for potential NBS-LRR genes in the *N. indica* genome using BLASTP (e-value = 1e−10). Then, based on the HMM model of NBS, HMMER v3.2.1 (http://hmmer.org/ , 10 January 2022, date last accessed) was used to identify NBS-LRR candidate genes. All candidate NBS-encoding genes from the above methods were integrated and submitted to the HMMER website (https://www.ebi.ac.uk/Tools/hmmer/search/hmmscan , 10 January 2022, date last accessed) to search the TIR, RPW8, CCX, NBS, and LRR domains.

All protein sequences of *A. thaliana* and *N. indica* were aligned to the NB-ARC1_ARC2 HMM^66^ of the NB-ARC domain using the hmmalign script from the HMMER v3.2.1 package (http://hmmer.org/ , 10 January 2022, date last accessed). Then we performed *Belvu*^67^ to trim the alignment to the NBS-ARC domain region and removed columns and sequences with over 95% gaps. Then, we manually verified the NB-ARC domain of the sequence alignments by Jalview.^68^ Finally, A maximum-likelihood phylogenetic tree was constructed using RAxML v8.2.12^69^ based on the JTT model for amino acid substitution, with other parameters following Kourelis et al.^65^ The gene cluster was defined with the criteria that in the range of up to 200 kb, non-NBS-LRR genes were restricted to eight within the cluster.^70^ TBtools v1.098^71^ was used to visualize the conserved domain of *N. indica* NBS-LRR proteins identified by MEME (http://meme-suite.org/tools/meme , 10 January 2022, date last accessed) (the maximum number of motifs set to 20) and to display the physical location of NBS genes in the *N. indica* genome.

All NBS protein sequences from *N. indica* were used to construct a phylogenetic tree following the method illustrated above. RNA-seq data from different tissues of *N. indica* were mapped back to the *N. indica* genome using Hisat2 v2.0.0^72^ with default settings. Then, the value of FPKM (fragments per kilobase of exon per million fragments) was calculated by StringTie v1.3.3^73^ and standardized using Log_2_ FPKM to quantify the expression level of NBS-LRR genes for each tissue. A heatmap of the gene expression profile was then drawn using the HeatMap tool of TBtools v1.098.^71^

## 3. Results and discussion

### 3.1. Genome assembly and annotation

We first generated ∼ 72.88 Gb Illumina paired-end reads (150 bp) (Supplementary Table S2). Based on the 17 *k-mer* analysis, the *N. indica* genome was estimated to be 620 Mb in size with a high heterozygous ratio of 1.10% (Supplementary Fig. S1). In addition, ∼ 141 Gb of PacBio sequencing subreads and ∼ 93 Gb of Hi-C reads were generated for draft genome assembly. They were corrected, trimmed, and assembled to preliminarily produce a 1.01 Gb genome that contained 1,891 contigs with a contig N50 of 2.39 Mb. After removing redundancy and polishing, we obtained a 520 Mb draft genome containing 283 contigs, with a contig N50 size of 7.17 Mb. We then anchored these contigs to nine pseudochromosomes using the Hi-C data, and 460 Mb (83.87%) of contigs were clustered and oriented into nine pseudochromosomes with sizes ranging from 66.87 Mb to 40.70 Mb (Supplementary Fig. S2 and Supplementary Table S3). The final chromosome-level assembly was 520 Mb in length with a scaffold N50 of 49.18 Mb, constituting 89.65% of the predicted genome size (Table 1). A total of 94.8% of the complete BUSCOs were found in the final assembly (Supplementary Table S4) and the Hi-C interactions plot shows a matrix with a clear plaid pattern indicating that the *N. indica* genome is nearly complete and of high quality (Supplementary Fig. S2).

**Table 1:** Statistics of the *N. indica* genome assembly and annotation

Feature	*N. indica*
Assembly	
Assembly size (Mb)	523
Number of contigs	283
Contig N50 (Mb)	7.17
Number of scaffolds	182
Scaffold N50 (Mb)	49.18
GC content (%)	37
Complete BUSCO (%)	94.8
Annotation	
Repeat region % of assembly	61.45
Protein-coding genes	29,983
Average gene length (bp)	6,473

By combining *de novo*, homology-based, and transcriptome-based approaches, a total of 29,983 protein-coding genes were predicted in the assembled genome with an average gene length of 6,473 bp (Supplementary Table S5). In total, 24,871 (82.9%) coding proteins were assigned functions by searching the eggNOG, InterPro, NR, and Swiss-Prot databases (Supplementary Table S6). Among these genes, 1,275 TFs belonging to 57 families were identified (Supplementary Table S7).

### 3.2. Repetitive content and recent LTR-RT expansion

**Figure 1:**
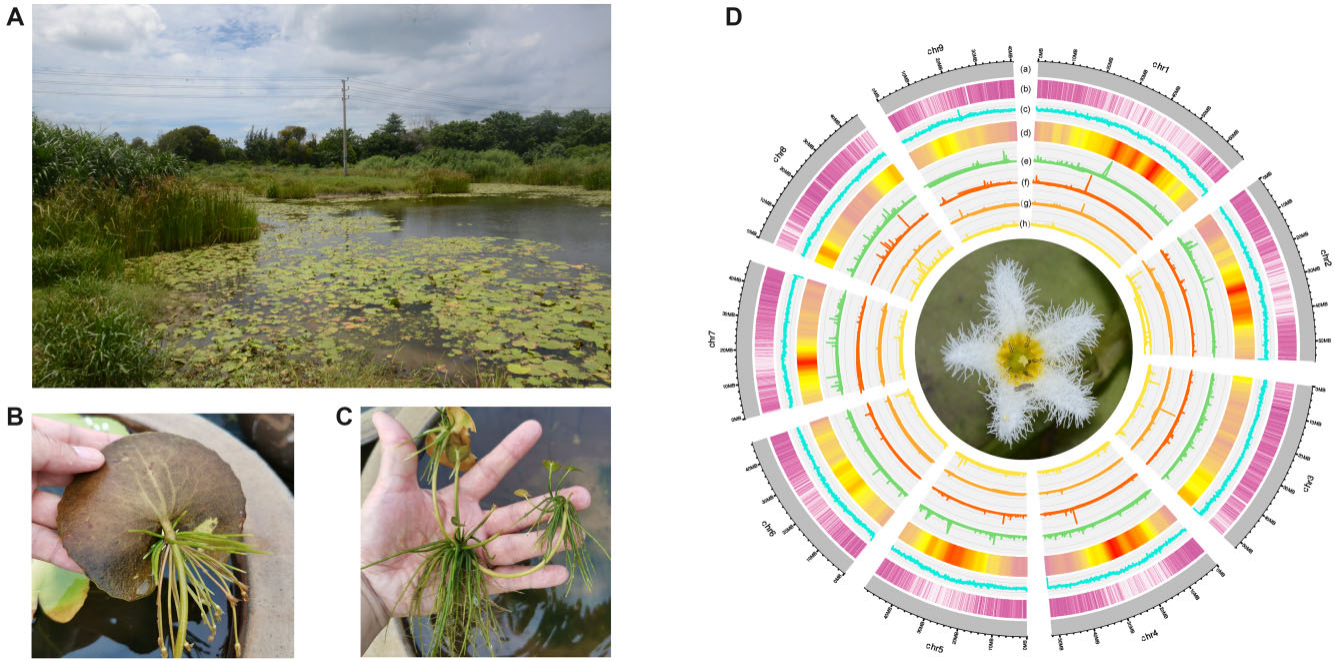
Characteristic of *N. indica* plant and genome. (A) The habitat of *N. indica*. (B) Leaves and associated adventitious roots of *N. indica*. (C), Vegetative reproduction of *N. indica*. (D) An overview of genomic features of *N. indica*. (a) Circular representation of the pseudomolecules (Mb), (b) gene density, (c) distribution of guanine–cytosine (GC) content, (d) LTR density, (e–h) expression of genes in different tissues (from outside to inside tacks: root, stem leaf and flower). All distributions are drawn in a window size of 1 Mb.

The overall integrity and accuracy of the *N. indica* genome have enabled a comprehensive analysis of TEs. By integrating homology-based and *de novo* approaches, we found that ∼ 61.45% of the assemblies were repetitive elements, and more than half (52.29%) of the *N. indica* genome consisted of TEs (Supplementary Table S8). Approximately 65% of TEs are LTR-RTs. Notably, the most abundant LTR-RTs family was *Gypsy*, occupying 41.36% of all LTR elements, followed by *Copia* at 24.31% (Supplementary Table S8). Most LTR-RTs accumulate around centromeres and pericentromeric regions in *N. indica*, which may reduce the impact directly compared with insertion into genes (Fig. 1). TEs have been confirmed to escape silencing and to experience a quick outburst.^74^ Previous studies have confirmed that TE insertions could produce new genetic variants and then promote speciation.^75–77^ Such as in *Oryza*, the rapid and recent evolution of LTR retrotransposons has been reported to drive the speciation and diversification of rice. They have led to the variation and evolution of the rice genome structure and played an important role in influencing the expression and functional differentiation of rice functional genes during the formation and diversity of rice species.^78^ Also, in the rye genome, the increased TE bursts contribute to the increase of transposed duplicated genes, such as starch pathway synthesis-related genes, in which new functional differentiation of genes has occurred, suggesting that TE-driven gene duplication can provide new resources for the generation of new functional differentiation of genes, and thus provide the necessary basis for the adaptive evolution and speciation.^79^ In addition, in Tibetan peach, SINE-type TEs were expanded and may play a crucial role in adapting to high-plateau environments and speciation.^80^ In conclusion, we here observed that LTR-RTs experienced a significant burst in the past 1 Mya (Supplementary Fig. S3), corresponding to the speciation time of *N. indica*. We inferred that TE mobilization during the burst might contribute to the speciation of *N. indica*.

However, as a ubiquitous component of eukaryotic genomes, the burst of LTR-RTs is an important force for plant genome expansion, and the removal of LTR-RTs can also cause rapid genome shrinkage.^81–83^ Unequal recombination (UR) to excise TEs is the general method for genome contraction, yielding solo-LTRs that reflect the rate of TE removal in a genome.^83^^,^^84^ In *N. indica*, relatively high removal rates 3.24 (S: 31,025, I: 9,565) were observed in *N. indica*, compared with *C. tinctorius* (S/I: 2.917; S: 78,664; I: 26,961) and *C. canephora* (S/I: 2.66; S: 13,800; I: 4,529). These results imply that the recent expansion of LTR-RTs in *N. indica* failed to facilitate genome size evolution. Moreover, the higher UR rate in *N. indica* may lead to genome downsizing.

**Figure 2:**
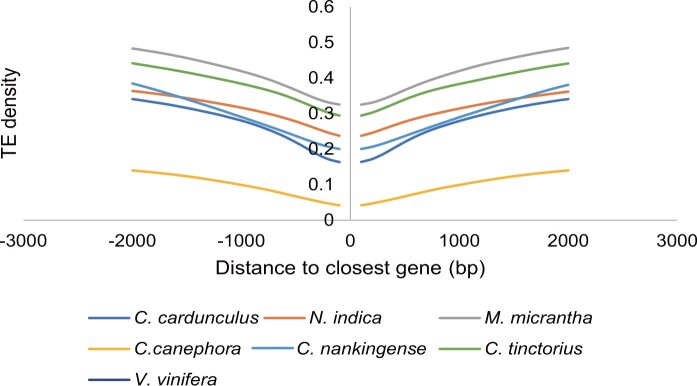
Comparison of TE density around the closest genes between species. Enrichment of TEs in 100-bp bins in the regions 2 kb upstream of the start codon and 2 kb downstream of the stop codon between all genes of *N. indica*, *C. tinctorius*, *C. cardunculus*, *C. nankingense*, *M. micrantha*, *V. vinifera*, and *C. canephora.* The *x* axis indicates the distance from TEs to the start codon (stop codon) of the closest gene; the *y* axis indicates the density of genes with TEs at different distances to the start codon (stop codon).

As an invasive species, rapid adaptation to a novel habitat is a critical prerequisite for successful invasion.^21^^,^^85^ TE insertions near genes influence the expression of neighbouring genes in response to environmental changes.^86^^,^^87^ For example, a *Copia*-type retrotransposon named *ONSEN* confers heat-mediated activation to nearby genes.^88^^,^^89^ Similarly, the tomato *Copia* LTR retrotransposon family *Rider* may confer drought-responsive expression to neighbouring genes.^90^ We explored whether the density of TEs located near genes differs between invasive and non-invasive plants. Our comparative analyses revealed that the density of TEs was higher in the upstream and downstream regions of genes in invasive species (*N. indica*, *C. tinctorius*, *C. cardunculus*, *C. nankingense*, and *M. micrantha*) than in non-invasive species (*V. vinifera* and *C. canephora*) (Fig. 2), suggesting that a relatively higher TE density may contribute to key adaptive variation in response to changing environments. Similar to previous reports, TEs are highly enriched in the wider-distribution species *Capsella rubella* compared with its outcrossing sister species *Capsella grandiflora*, suggesting that TEs can potentially contribute to adapting to novel environments that experience a genetic bottleneck.^22^

Additionally, LTRs can also confer stress-inducible expression patterns on their flanking TFs, such as *Ruby*, an MYB transcriptional activator of anthocyanin production in *Citrus sinensis*, which is regulated by a nearby *Copia*-like retrotransposon under cold induction.^91^ In *N. indica*, as the key regulator of abiotic stresses, APETALA2/ETHYLENE RESPONSIVE FACTOR (AP2/ERF) family TFs are located in LTR-rich regions compared with the other TFs, indicating the potential impact of LTR-RTs on neighbouring AP2/ERF TFs (Supplementary Fig. S4). It can be inferred that TEs might influence the expression of AP2/ERF TFs in response to environmental stress during the rapid adaptation of *N. indica*.

### 3.3. Genome evolution and expansion of gene families

**Figure 3:**
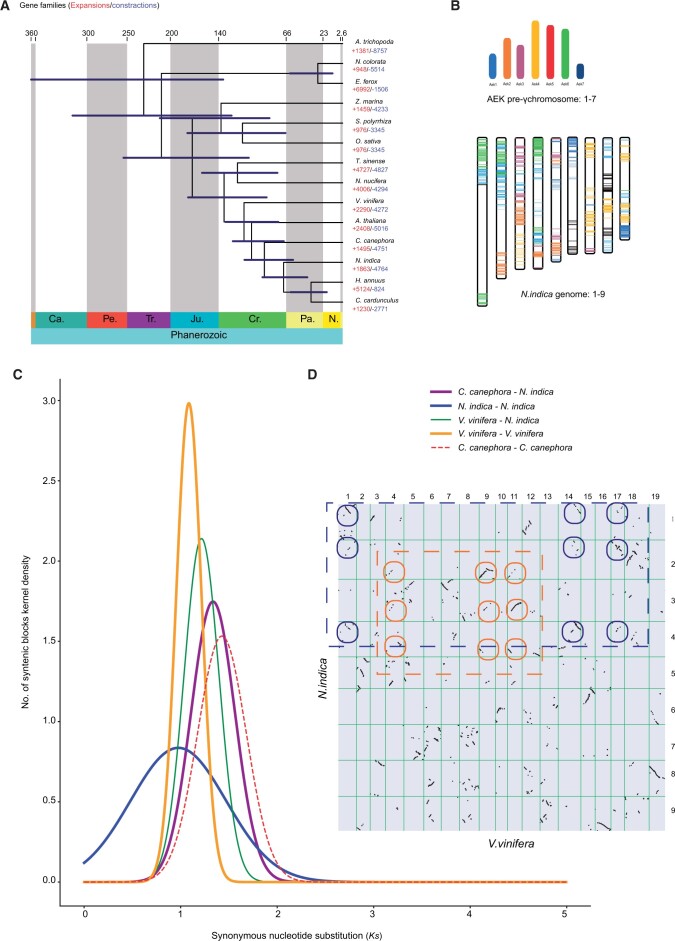
Evolutionary history of *N. indica*. (a) Phylogenetic tree of 14 plant species and the evolution of gene families. The divergence times were estimated using MCMCTree and indicated by light blue bars at the internodes with a 95% highest posterior density. Cr: Cretaceous; Ju: Jurassic; Pa: Paleogene; Ne: Neogene. The numbers of gene-family expansions and contractions are indicated by red and blue numbers. (b) Comparison with ancestral eudicot karyotype (AEK) chromosomes reveals synteny. The syntenic AEK blocks are painted onto *N. indica* chromosomes. (c) *Ks* distributions of orthologous and paralogous genes among *C. canephora, V. vinifera*, and *N. indica*. (d) Comparison of *N. indica* and *V. vinifera* genomes. Dot plots of orthologs showing a 3–3 chromosomal relationship between the *N. indica* genome and *V. vinifera* genome.

A phylogenomic tree with estimated divergence times for the 14 species was constructed based on a concatenated sequence alignment of 230 common single-copy genes. Our phylogenetic results revealed a close relationship between *N. indica* and Asteraceae, which diverged from the most recent common ancestor of *C. cardunculus* and *H. annuus* at ∼ 68 Mya, after separating *V. vinifera* at ∼ 91.5 Mya (Fig. 3). Based on sequence homology, a total of 389,445 (88.3%) genes were clustered into 29,220 orthogroups, 11,678 of which were species-specific orthogroups and 6,885 gene families were shared by all 14 species. In *N. indica*, 27,286 protein-coding genes were assigned to 12,600 families and 926 gene families containing 4,249 genes were *N. indica-*specific (Supplementary Table S9). GO and KEGG enrichment analysis showed that these unique gene families were enriched for GO terms, such as ‘GO : 0009553, embryo sac development’, ‘GO : 0000741, karyogamy’ and ‘GO : 0004869, cysteine-type endopeptidase inhibitor activity’ and KEGG categories such as ‘ko00750, Vitamin B6 metabolism’ and ‘ko01040, Biosynthesis of unsaturated fatty acids’ (Supplementary Tables S10 and S11). Comparative genomic analysis showed that 2,055 gene families were expanded in *N. indica*, whereas 4,777 were contracted. The expanded gene families of *N. indica* were significantly enriched in catalytic activity, brassinosteroid biosynthesis, and plant–pathogen interactions (Supplementary Tables S12 and S13). In addition, rapidly expanded gene families showed high enrichment in response to hypoxia, decreased oxygen levels, and plant–pathogen interactions (Supplementary Tables S14 and S15). We observed that the process of plant–pathogen interaction was enriched in rapidly expanded gene families of *N. indica* (Supplementary Table S15). This may explain why *N. indica* adapts quickly to diverse environments and is widely distributed in neotropical regions.^2^

To further understand the paleohistory of the *N. indica* genome, we analysed WGD events. Dot plots of orthologs between *N. indica* and *V. vinifera* showed a 3–3 orthology ratio (Fig. 3), indicating a similar evolutionary history between the genomes of *N. indica* and *V. vinifera.* The *Ks* distribution of the intragenomic paralogs in the *N. indica* genome showed a single peak at *Ks* values of ∼ 0.98, which was close to the peak of *V. vinifera* (1.21) and *C. canephora* (1.08) (Fig. 3). This clearly indicated that these three species may have experienced a shared WGD event in their common ancestor. Given that the *V. vinifera* and *C. canephora* genomes have been confirmed to experience only an ancient whole-genome triplication (WGT) event,^92^^,^^93^*N. indica* likely experienced this WGT event shared by the recent common ancestor of core eudicots.^94^ Furthermore, intragenomic syntenic analysis of *N. indica* (1–3 chromosomal relationships) revealed a WGT event (Supplementary Fig. S5). We also compared the syntenic depth ratios of *N. indica*, *V. vinifera*, and *C. canephora*. All paired genome comparisons detected an identical 2:2 syntenic depth ratio (Supplementary Figs. S6–S8). The evidence provided here is sufficient to prove that the *N. indica* genome experienced only the gamma (γ) WGT event shared by all core eudicots.

WGDs result in the complete doubling of the genome and produce abundant duplicated genes to facilitate adaptation to changing environments.^95^ We performed GO and KEGG enrichment analyses of the duplicated genes generated by WGT-γ. These WGT-related gene pairs were significantly enriched in some processes that are essential for growth and development, such as nucleobase-containing compound biosynthetic process, regulation of nucleic acid-templated transcription, carbon fixation in photosynthetic organisms, and regulation of gene expression and biosynthetic process (Supplementary Tables S16 and S17). This is consistent with previous reports on *M. micrantha*; only genes that are crucial for survival were retained during the evolution procedure, whereas redundant genes were lost.^26^ Moreover, in comparison with the ancestral eudicot karyotype genome, only 4,154 (13.85%) genes were identified in *N. indica*, lower than 6,828 (25.9%) genes in *V. vinifera*, and 13,932 (34.9%) in *C. tinctorius*.^96^ Despite a lack of recent WGDs, 41% of the genes were duplicated through dispersed duplications. A substantial number of dispersed duplicates may have been generated after the core-eudicot γ triplication, and they may contribute to phenotypic diversification, such as stress-resistance traits.^97^ In the present study, a large number of dispersed duplicates were detected in the *N. indica* genome, suggesting multiple chromosome rearrangements that occurred after the WGT-γ event, which may be a critical factor in the success of *N. indica* colonization of a novel environment.

### 3.4. Analysis of NBS-LRR gene family

**Figure 4:**
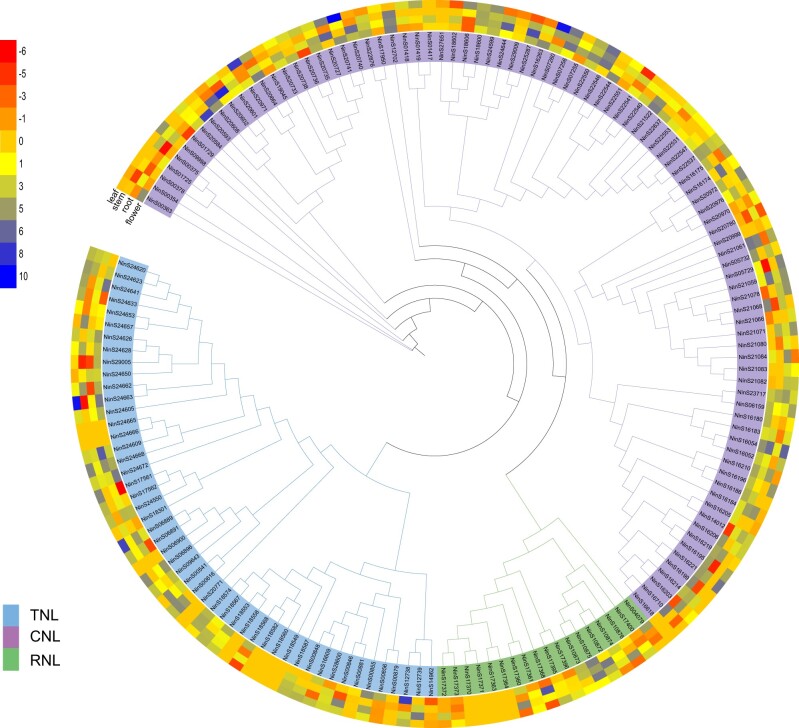
NBS-LRR gene family in *N. indica*. Phylogenetic reconstruction of the NBS-LRR proteins (160) in *N. indica*. The colour scale represents FPKM normalized log2-transformed counts. All of these proteins were grouped into four clades. Blue, green, yellow, and orange correspond to CNL, TNL, RNL, and ANL subfamilies, respectively. Heatmap showing the differential expression of NBS-LRR genes according to the transcriptome data from leaf, flower, stem, and root. The tree only shows bootstrap values >70%.

RefPlantNLR^65^ is an accurate and comprehensive database, including 481 NLRs grouped in 31 genera and 11 orders of angiosperms. In this database, NBS-LRR were divided into four clades, TIR-NLRs, CC_R_-NLRs, CC_G10_-NLRs (containing autoactive coiled-coil domains; hereafter termed ANLs) and CC-NLRs. According to the RefPlantNLR database,^65^ phylogenetic trees, and domain architectures (Fig. 4 and Supplementary Figs. S9 and S10), 160 NBS-LRR genes were identified in the *N. indica* genome and clustered into four groups, CC-NBS-LRR (CNL) (68), TIR-NBS-LRR (TNL) (50), CC_G10_-NBS-LRR (ancient and autonomous NLRs, ANL) (24), and RPW8-NBS-LRR (RNL) (18) (Supplementary Tables S18 and S19). Similar to the previous study, the ANL subclass is a monophyletic group that is distinguished from the other CNL clades.^65^^,^^98^ In addition, all NBS-LRR genes were unevenly distributed across all nine chromosomes (Supplementary Fig. S11). Previous studies have revealed that a substantial number of NBS-LRR genes are clustered across chromosomes and are associated with tandem duplication.^99^^,^^100^ In *N. indica*, 26 gene clusters containing 131 NBS-LRR genes were identified. Additionally, NBS-LRR genes located in the same cluster showed a close relationship in the phylogenetic tree, indicating that tandem duplication is responsible for NBS-LRR gene expansion in *N*. *indica*. The expression patterns of the NBS-LRR genes in the four tissues were significantly different in *N. indica*. The leaves had 44 NBS-LRR genes that exhibited higher expression levels than other tissues (Fig. 4 and Supplementary Table 19), which can explain why leaves come into contact with water to resist pathogens. It has been reported that many aquatic plant genomes, such as *S. polyrhiza*, *Z. marina*, and *Utricularia gibba*, have convergently lost the majority of their NLR genes and well-known downstream immune signalling complex ENHANCED DISEASE SUSCEPTIBILITY 1 (*EDS1*)*/*PHYTOALEXIN DEFICIENT 4 (*PAD4*).^101^ On the contrary, we identified several homologs of known immunity genes in the aquatic plant *N. indica* (Supplementary Table 20), such as *EDS1*, *PAD4*, and SENESCENCE ASSOCIATED GENE 101 (*SAG101*), which indicated that signalling components are likely conserved in *N. indica* and these genes were lost independently in different lineages.^101^ In addition, our results well supported the view that the loss of *EDS1*/*PAD4*, might be limited to a few species with a low number of NLR genes.^101^

## 4. Conclusions

We assembled and annotated a chromosome-scale reference genome of *N. indica* using PacBio, Illumina, and Hi-C sequencing data, which will provide an opportunity to explore the potential mechanisms underlying its invasiveness. Our study highlights the important role of TEs in the rapid invasion of *N. indica* in response to environmental change, colonization, and expansion as invasive species. Based on the gene family analysis, the expanded gene families were significantly enriched in the function of plant–pathogen interaction and rapidly expanded gene families were enriched in response to hypoxia, decreased oxygen levels, and plant–pathogen interaction, which may coincide with the adaptation of *N. indica* to the low oxygen level in semi-arid land as well as the eutrophic body of water. Furthermore, we identified the NBS-LRR gene family, which will be valuable in future evolutionary and functional studies. As the first sequenced species in Menyanthaceae, our results provide insights into the evolutionary origin and ecological control of *Nymphoides*.

## Supplementary Material

dsac022_Supplementary_Data

## Data Availability

All the data sets used in this study are available at the China National GeneBank DataBase (CNGBdb, https://db.cngb.org/) website under the accessions CNS0525744-CNS0525745 and CNS0548186-CNS0548187 with CNGB-Project ID CNP0002767.

## Author details

J.-M.C. and Z.-Z.L. designed the study and led the research. J.-S.Y. wrote the draft manuscript. J.-S.Y., Z.-H.Q., and T.S. contributed to the genome assembly and annotation. J.-S.Y., and Z.-H.Q. participated in genome evolution analysis. J.-M.C., T.S., and Z.-Z.L. contributed substantially to the revisions. The final manuscript has been read and approved by all authors.

## References

[1] Ornduff R. 1966, The origin of dioecism from heterostyly in Nymphoides (Menyanthaceae), Evolution, 20, 309–14. 28562972 10.1111/j.1558-5646.1966.tb03368.x

[2] Tippery N.P. , Les D.H. 2011, Phylogenetic relationships and morphological evolution in Nymphoides (Menyanthaceae), Syst. Bot., 36, 1101–13.

[3] Tippery N.P. , Sears N.L., Zentner A.B., Sivadas V. 2018, Evidence for allopolyploid speciation in Nymphoides (Menyanthaceae), Syst. Bot., 43, 117–29.

[4] Shibayama Y. , Kadono Y. 2003, Floral morph composition and pollen limitation in the seed set of *Nymphoides indica* populations, Ecol. Res., 18, 725–37.

[5] Shibayama Y. , Kadono Y. 2007, Reproductive success and genetic structure of populations of the heterostylous aquatic plant *Nymphoides indica* (L.) Kuntze (Menyanthaceae), Aquat. Bot., 86, 1–8.

[6] Saunders K. 2005, First record of Nymphoides *i*ndica (Menyanthaceae) in Texas. SIDA Contrib Bot., 21, 2441–3.

[7] USDA-APHIS. 2012, U.S. Department of Agriculture Animal and Plant Health Inspection Service. https://usdasearch.usda.gov/search?utf8=%3F&affiliate=usda-aphis&query=Nymphoides&commit=Search (10 January 2022, date last accessed).

[8] NCDA (North Carolina Department of Agriculture). 2017, Electronic database. http://www.ncagr.gov/plantindustry/plant/weed/noxweed.htm (10 January 2022, date last accessed).

[9] Champion P.D. , Clayton J.S. 2003, The evaluation and management of aquatic weeds in New Zealand. In Plant Invasions: Ecological Threats and Management Solutions, pp. 429–34. Backhuys Publishers: Leiden, the Netherlands.

[10] Larson D. 2007, Growth of three submerged plants below different densities of Nymphoides peltata (S. G. Gmel.) Kuntze, Aquat. Bot., 86, 280–4.

[11] GISD (Global Invasive Species Database). 2022, Species profile: *Nymphoides peltata*. http://www.iucngisd.org/gisd/species.php?sc=225 (10 January 2022, date last accessed).

[12] Marwat S.K. , Khan M.A., Fazal-ur-Rehman Ahmad M., Zafar M. 2011, Biodiversity and importance of floating weeds of Dara Ismail, Khan District of KPK, Pakistan, Afr J. Tradit. Complement. Altern. Med., 8, 97–107. 22754062 10.4314/ajtcam.v8i5S.17 PMC3252727

[13] Schultz R. , Dibble E. 2012, Effects of invasive macrophytes on freshwater fish and macroinvertebrate communities: the role of invasive plant traits, Hydrobiologia, 684, 1–14.

[14] Vanneste K. , Baele G., Maere S., Van de Peer Y. 2014, Analysis of 41 plant genomes supports a wave of successful genome duplications in association with the Cretaceous-Paleogene boundary, Genome Res., 24, 1334–47. 24835588 10.1101/gr.168997.113 PMC4120086

[15] Ren R. , Wang H., Guo C., et al 2018, Widespread whole genome duplications contribute to genome complexity and species diversity in angiosperms, Mol. Plant., 11, 414–28. 29317285 10.1016/j.molp.2018.01.002

[16] Soltis P.S. , Soltis D.E. 2016, Ancient WGD events as drivers of key innovations in angiosperms, Curr. Opin. Plant Biol., 30, 159–65. 27064530 10.1016/j.pbi.2016.03.015

[17] Jiao Y. 2018, Double the genome, double the fun: genome duplications in Angiosperms, Mol. Plant., 11, 357–8. 29476919 10.1016/j.molp.2018.02.009

[18] Zhang L. , Wu S., Chang X., et al 2020, The ancient wave of polyploidization events in flowering plants and their facilitated adaptation to environmental stress, Plant. Cell Environ., 43, 2847–56. 33001478 10.1111/pce.13898

[19] Qiu Y. , Köhler C. 2020, Mobility connects: transposable elements wire new transcriptional networks by transferring transcription factor binding motifs, Biochem. Soc. Trans., 48, 1005–17. 32573687 10.1042/BST20190937 PMC7329337

[20] Zhang Y. , Li Z., Zhang Y., et al 2021, Evolutionary rewiring of the wheat transcriptional regulatory network by lineage-specific transposable elements, Genome Res., 31, 2276–89. 10.1101/gr.275658.121 PMC8647832 34503979

[21] Casacuberta E. , González J. 2013, The impact of transposable elements in environmental adaptation, Mol. Ecol., 22, 1503–17. 23293987 10.1111/mec.12170

[22] Niu X.M. , Xu Y.C., Li Z.W., et al 2019, Transposable elements drive rapid phenotypic variation in *Capsella rubella*, Proc. Natl. Acad. Sci. U S A, 116, 6908–13. 30877258 10.1073/pnas.1811498116 PMC6452725

[23] McHale L. , Tan X., Koehl P., Michelmore R.W. 2006, Plant NBS-LRR proteins: adaptable guards, Genome Biol., 7, 212. 16677430 10.1186/gb-2006-7-4-212 PMC1557992

[24] Shao Z.Q. , Xue J.Y., Wang Q., Wang B., Chen J.Q. 2019, Revisiting the origin of plant NBS-LRR genes, Trends Plant Sci., 24, 9–12. 30446304 10.1016/j.tplants.2018.10.015

[25] Peng Y. , Lai Z., Lane T., et al 2014, De novo genome assembly of the economically important weed horseweed using integrated data from multiple sequencing platforms, Plant Physiol., 166, 1241–54. 25209985 10.1104/pp.114.247668 PMC4226366

[26] Liu B. , Yan J., Li W., et al 2020, *Mikania micrantha* genome provides insights into the molecular mechanism of rapid growth, Nat. Commun., 11, 340. 31953413 10.1038/s41467-019-13926-4 PMC6969026

[27] Ramachandran D. , Huebner C.D., Daly M., Haimovitz J., Swale T., Barrett C.F. 2021, Chromosome level genome assembly and annotation of highly invasive Japanese stiltgrass (*Microstegium vimineum*), Genome Biol. Evol., 13, evab238. 34718556 10.1093/gbe/evab238 PMC8598173

[28] Oh D.H. , Kowalski K.P., Quach Q.N., et al 2022, Novel genome characteristics contribute to the invasiveness of *Phragmites australis* (common reed), Mol. Ecol., 31, 1142–59. 34839548 10.1111/mec.16293 PMC9300010

[29] Li S.P. , Hsieh T.H., Lin C.C. 2002, The genus *Nymphoides Séguier* (Menyanthaceae) in Taiwan, Taiwania, 47, 246–58.

[30] Bolger A.M. , Lohse M., Usadel B. 2014, Trimmomatic: a flexible trimmer for illumina sequence data, Bioinformatics, 30, 2114–20. 24695404 10.1093/bioinformatics/btu170 PMC4103590

[31] Marçais G. , Kingsford C. 2011, A fast, lock-free approach for efficient parallel counting of occurrences of k-mers, Bioinformatics, 27, 764–70. 21217122 10.1093/bioinformatics/btr011 PMC3051319

[32] Liu B. , Shi Y.J., Yuan J.Y., et al 2013, Estimation of genomic characteristics by analyzing k-mer frequency in de novo genome projects, arXiv, 1308.

[33] Koren S. , Walenz B.P., Berlin K., Miller J.R., Bergman N.H., Phillippy A.M. 2017, Canu: scalable and accurate long-read assembly via adaptive k-mer weighting and repeat separation, Genome Res., 27, 722–36. 28298431 10.1101/gr.215087.116 PMC5411767

[34] Hu J. , Fan J., Sun Z., Liu S. 2020, NextPolish: a fast and efficient genome polishing tool for long-read assembly, Bioinformatics, 36, 2253–5. 31778144 10.1093/bioinformatics/btz891

[35] Roach M.J. , Schmidt S.A., Borneman A.R. 2018, Purge Haplotigs: allelic contig reassignment for third-gen diploid genome assemblies, BMC Bioinformatics, 19, 460. 30497373 10.1186/s12859-018-2485-7 PMC6267036

[36] Servant N. , Varoquaux N., Lajoie B.R., et al 2015, HiC-Pro: an optimized and flexible pipeline for Hi-C data processing, Genome Biol., 16, 259. 26619908 10.1186/s13059-015-0831-x PMC4665391

[37] Durand N.C. , Shamim M.S., Machol I., et al 2016, Juicer provides a one-click system for analyzing loop-resolution Hi-C experiments, Cell Syst., 3, 95–8. 27467249 10.1016/j.cels.2016.07.002 PMC5846465

[38] Dudchenko O. , Batra S.S., Omer A.D., et al 2017, De novo assembly of the *Aedes aegypti* genome using Hi-C yields chromosome-length scaffolds, Science, 356, 92–5. 28336562 10.1126/science.aal3327 PMC5635820

[39] Durand N.C. , Robinson J.T., Shamim M.S., et al 2016, Juicebox provides a visualization system for Hi-C contact maps with unlimited zoom, Cell Syst., 3, 99–101. 27467250 10.1016/j.cels.2015.07.012 PMC5596920

[40] Simão F.A. , Waterhouse R.M., Ioannidis P., Kriventseva E.V., Zdobnov E.M. 2015, BUSCO: assessing genome assembly and annotation completeness with single-copy orthologs, Bioinformatics, 31, 3210–2. 26059717 10.1093/bioinformatics/btv351

[41] Bao W. , Kojima K.K., Kohany O. 2015, Repbase Update, a database of repetitive elements in eukaryotic genomes, Mob. DNA, 6, 11. 26045719 10.1186/s13100-015-0041-9 PMC4455052

[42] Stanke M. , Keller O., Gunduz I., Hayes A., Waack S., Morgenstern B. 2006, AUGUSTUS: ab initio prediction of alternative transcripts, Nucleic Acids Res., 34, W435–9. 16845043 10.1093/nar/gkl200 PMC1538822

[43] Birney E. , Durbin R. 2000, Using GeneWise in the *Drosophila* annotation experiment, Genome Res., 10, 547–8. 10779496 10.1101/gr.10.4.547 PMC310858

[44] Grabherr M.G. , Haas B.J., Yassour M., et al 2011, Full-length transcriptome assembly from RNA-Seq data without a reference genome, Nat. Biotechnol., 29, 644–52. 21572440 10.1038/nbt.1883 PMC3571712

[45] Haas B.J. , Delcher A.L., Mount S.M., et al 2003, Improving the Arabidopsis genome annotation using maximal transcript alignment assemblies, Nucleic Acids Res., 31, 5654–66. 14500829 10.1093/nar/gkg770 PMC206470

[46] Haas B.J. , Salzberg S.L., Zhu W., et al 2008, Automated eukaryotic gene structure annotation using EVidenceModeler and the Program to Assemble Spliced Alignments, Genome Biol., 9, R7. 18190707 10.1186/gb-2008-9-1-r7 PMC2395244

[47] Huerta-Cepas J. , Szklarczyk D., Heller D., et al 2019, eggNOG 5.0: a hierarchical, functionally and phylogenetically annotated orthology resource based on 5090 organisms and 2502 viruses, Nucleic Acids Res., 47, D309–14. 30418610 10.1093/nar/gky1085 PMC6324079

[48] Cantalapiedra C.P. , Hernández-Plaza A., Letunic I., Bork P., Huerta-Cepas J. 2021, eggNOG-mapper v2: functional annotation, orthology assignments, and domain prediction at the metagenomic scale, Mol. Biol. Evol., 38, 5825–9. 34597405 10.1093/molbev/msab293 PMC8662613

[49] Zdobnov E.M. , Apweiler R. 2001, InterProScan–an integration platform for the signature-recognition methods in InterPro, Bioinformatics, 17, 847–8. 11590104 10.1093/bioinformatics/17.9.847

[50] Tian F. , Yang D.C., Meng Y.Q., Jin J., Gao G. 2020, PlantRegMap: charting functional regulatory maps in plants, Nucleic Acids Res., 48, D1104–13. 31701126 10.1093/nar/gkz1020 PMC7145545

[51] Ou S. , Su W., Liao Y., et al 2019, Benchmarking transposable element annotation methods for creation of a streamlined, comprehensive pipeline, Genome Biol., 20, 275. 31843001 10.1186/s13059-019-1905-y PMC6913007

[52] Ou S. , Jiang N. 2018, LTR_retriever: a highly accurate and sensitive program for identification of long terminal repeat retrotransposons, Plant Physiol., 176, 1410–22. 29233850 10.1104/pp.17.01310 PMC5813529

[53] Ossowski S. , Schneeberger K., Lucas-Lledó J.I., et al 2010, The rate and molecular spectrum of spontaneous mutations in *Arabidopsis thaliana*, Science, 327, 92–4. 20044577 10.1126/science.1180677 PMC3878865

[54] Emms D.M. , Kelly S. 2019, OrthoFinder: phylogenetic orthology inference for comparative genomics, Genome Biol., 20, 238. 31727128 10.1186/s13059-019-1832-y PMC6857279

[55] Edgar R.C. 2004, MUSCLE: a multiple sequence alignment method with reduced time and space complexity, BMC Bioinform., 5, 113. 10.1186/1471-2105-5-113 PMC517706 15318951

[56] Suyama M. , Torrents D., Bork P. 2006, PAL2NAL: robust conversion of protein sequence alignments into the corresponding codon alignments, Nucleic Acids Res., 34, W609–12. 16845082 10.1093/nar/gkl315 PMC1538804

[57] Talavera G. , Castresana J. 2007, Improvement of phylogenies after removing divergent and ambiguously aligned blocks from protein sequence alignments, Syst. Biol., 56, 564–77. 17654362 10.1080/10635150701472164

[58] Nguyen L.T. , Schmidt H.A., von Haeseler A., Minh B.Q. 2015, IQ-TREE: a fast and effective stochastic algorithm for estimating maximum-likelihood phylogenies, Mol. Biol. Evol., 32, 268–74. 25371430 10.1093/molbev/msu300 PMC4271533

[59] Yang Z. 2007, PAML 4: phylogenetic analysis by maximum likelihood, Mol. Biol. Evol., 24, 1586–91. 17483113 10.1093/molbev/msm088

[60] Barreda V.D. , Palazzesi L., Tellería M.C., Olivero E.B., Raine J.I., Forest F. 2015, Early evolution of the angiosperm clade Asteraceae in the Cretaceous of Antarctica, Proc. Natl. Acad. Sci. U S A, 112, 10989–94. 26261324 10.1073/pnas.1423653112 PMC4568267

[61] Chaw S.M. , Chang C.C., Chen H.L., Li W.H. 2004, Dating the monocot-dicot divergence and the origin of core eudicots using whole chloroplast genomes, J. Mol. Evol., 58, 424–41. 15114421 10.1007/s00239-003-2564-9

[62] De Bie T. , Cristianini N., Demuth J.P., Hahn M.W. 2006, CAFE: a computational tool for the study of gene family evolution, Bioinformatics, 22, 1269–71. 16543274 10.1093/bioinformatics/btl097

[63] Wang Y. , Tang H., Debarry J.D., et al 2012, MCScanX: a toolkit for detection and evolutionary analysis of gene synteny and collinearity, Nucleic Acids Res., 40, e49. 22217600 10.1093/nar/gkr1293 PMC3326336

[64] Sun P. , Jiao B.B., Yang Y.Z., et al 2021, WGDI: a user-friendly toolkit for evolutionary analyses of whole-genome duplications and ancestral karyotypes. https://www.biorxiv.org/content/10.1101/2021.04.29.441969v1.abstract (10 January 2022, date last accessed). 10.1016/j.molp.2022.10.018 36307977

[65] Kourelis J. , Sakai T., Adachi H., Kamoun S. 2021, RefPlantNLR is a comprehensive collection of experimentally validated plant disease resistance proteins from the NLR family, PLoS Biol., 19, e3001124. 34669691 10.1371/journal.pbio.3001124 PMC8559963

[66] Bailey P.C. , Schudoma C., Jackson W., et al 2018, Dominant integration locus drives continuous diversification of plant immune receptors with exogenous domain fusions, Genome Biol., 19, 23. 29458393 10.1186/s13059-018-1392-6 PMC5819176

[67] Barson G. , Griffiths E. 2016, SeqTools: visual tools for manual analysis of sequence alignments, BMC Res. Notes, 9, 39. 26801397 10.1186/s13104-016-1847-3 PMC4724122

[68] Waterhouse A.M. , Procter J.B., Martin D.M., Clamp M., Barton G.J. 2009, Jalview Version 2–a multiple sequence alignment editor and analysis workbench, Bioinformatics, 25, 1189–91. 19151095 10.1093/bioinformatics/btp033 PMC2672624

[69] Stamatakis A. 2014, RAxML version 8: a tool for phylogenetic analysis and post-analysis of large phylogenies, Bioinformatics, 30, 1312–3. 24451623 10.1093/bioinformatics/btu033 PMC3998144

[70] Andersen E.J. , Ali S., Reese R.N., Yen Y., Neupane S., Nepal M.P. 2016, Diversity and evolution of disease resistance genes in Barley, Evol. Bioinform. Online, 12, 99–108. 27168720 10.4137/EBO.S38085 PMC4857794

[71] Chen C. , Chen H., Zhang Y., et al 2020, TBtools: an integrative toolkit developed for interactive analyses of big biological data, Mol. Plant., 13, 1194–202. 32585190 10.1016/j.molp.2020.06.009

[72] Kim D. , Paggi J.M., Park C., Bennett C., Salzberg S.L. 2019, Graph-based genome alignment and genotyping with HISAT2 and HISAT-genotype, Nat. Biotechnol., 37, 907–15. 31375807 10.1038/s41587-019-0201-4 PMC7605509

[73] Pertea M. , Pertea G.M., Antonescu C.M., Chang T.C., Mendell J.T., Salzberg S.L. 2015, StringTie enables improved reconstruction of a transcriptome from RNA-seq reads, Nat. Biotechnol., 33, 290–5. 25690850 10.1038/nbt.3122 PMC4643835

[74] Wicker T. , Gundlach H., Spannagl M., et al; International Wheat Genome Sequencing Consortium. 2018, Impact of transposable elements on genome structure and evolution in bread wheat, Genome Biol., 19, 1–18. 30115100 10.1186/s13059-018-1479-0 PMC6097303

[75] Lisch D. 2013, How important are transposons for plant evolution? Nat. Rev. Genet., 14, 49–61. 23247435 10.1038/nrg3374

[76] Serrato-Capuchina A. , Matute D.R. 2018, The role of transposable elements in speciation, Genes, 9, 254. 10.3390/genes9050254 PMC5977194 29762547

[77] Auvinet J. , Graça P., Belkadi L. 2018, Mobilization of retrotransposons as a cause of chromosomal diversification and rapid speciation: the case for the Antarctic teleost genus Trematomus, BMC Genom., 19, 339. 10.1186/s12864-018-4714-x PMC5941688 29739320

[78] Zhang Q.J. , Gao L.Z. 2017, Rapid and recent evolution of LTR retrotransposons drives rice genome evolution during the speciation of AA-genome Oryza species, G3 (Bethesda), 7, 1875–85. 28413161 10.1534/g3.116.037572 PMC5473765

[79] Li G. , Wang L., Yang J., et al 2021, A high-quality genome assembly highlights rye genomic characteristics and agronomically important genes, Nat. Genet., 53, 574–84. 33737755 10.1038/s41588-021-00808-z PMC8035075

[80] Wang X. , Liu S., Zuo H., et al 2021, Genomic basis of high-altitude adaptation in Tibetan Prunus fruit trees, Curr. Biol., 31, 3848–60. 34314676 10.1016/j.cub.2021.06.062

[81] Vitte C. , Panaud O. 2005, LTR retrotransposons and flowering plant genome size: emergence of the increase/decrease model, Cytogenet. Genome Res., 110, 91–107. 16093661 10.1159/000084941

[82] Zedek F. , Smerda J., Smarda P., Bureš P. 2010, Correlated evolution of LTR retrotransposons and genome size in the genus *Eleocharis*, BMC Plant Biol., 10, 265. 21118487 10.1186/1471-2229-10-265 PMC3095338

[83] Cossu R.M. , Casola C., Giacomello S., Vidalis A., Scofield D.G., Zuccolo A. 2017, LTR retrotransposons show low levels of unequal recombination and high rates of intraelement gene conversion in large plant genomes, Genome Biol. Evol., 9, 3449–62. 29228262 10.1093/gbe/evx260 PMC5751070

[84] Tian Z. , Rizzon C., Du J., et al 2009, Do genetic recombination and gene density shape the pattern of DNA elimination in rice long terminal repeat retrotransposons?, Genome Res., 19, 2221–30. 19789376 10.1101/gr.083899.108 PMC2792168

[85] Stapley J. , Santure A.W., Dennis S.R. 2015, Transposable elements as agents of rapid adaptation may explain the genetic paradox of invasive species, Mol. Ecol., 24, 2241–52. 25611725 10.1111/mec.13089

[86] Makarevitch I. , Waters A.J., West P.T., et al 2015, Transposable elements contribute to activation of maize genes in response to abiotic stress, PLoS Genet., 11, e1004915. 25569788 10.1371/journal.pgen.1004915 PMC4287451

[87] Hirsch C.D. , Springer N.M. 2017, Transposable element influences on gene expression in plants, Biochim. Biophys. Acta. Gene Regul. Mech., 1860, 157–65. 27235540 10.1016/j.bbagrm.2016.05.010

[88] Ito H. , Gaubert H., Bucher E., Mirouze M., Vaillant I., Paszkowski J. 2011, An siRNA pathway prevents transgenerational retrotransposition in plants subjected to stress, Nature, 472, 115–9. 21399627 10.1038/nature09861

[89] Cavrak V.V. , Lettner N., Jamge S., Kosarewicz A., Bayer L.M., Mittelsten Scheid O. 2014, How a retrotransposon exploits the plant’s heat stress response for its activation, PLoS Genet., 10, e1004115. 24497839 10.1371/journal.pgen.1004115 PMC3907296

[90] Benoit M. , Drost H.G., Catoni M., et al 2019, Environmental and epigenetic regulation of Rider retrotransposons in tomato, PLoS Genet., 15, e1008370. 31525177 10.1371/journal.pgen.1008370 PMC6762207

[91] Butelli E. , Licciardello C., Zhang Y., et al 2012, Retrotransposons control fruit-specific, cold-dependent accumulation of anthocyanins in blood oranges, Plant Cell, 24, 1242–55. 22427337 10.1105/tpc.111.095232 PMC3336134

[92] Jaillon O. , Aury J.M., Noel B., et al 2007, The grapevine genome sequence suggests ancestral hexaploidization in major angiosperm phyla, Nature, 449, 463–7. 17721507 10.1038/nature06148

[93] Denoeud F. , Carretero-Paulet L., Dereeper A., et al 2014, The coffee genome provides insight into the convergent evolution of caffeine biosynthesis, Science, 345, 1181–4. 25190796 10.1126/science.1255274

[94] Jiao Y. , Leebens-Mack J., Ayyampalayam S., et al 2012, A genome triplication associated with early diversification of the core eudicots, Genome Biol., 13, R3. 22280555 10.1186/gb-2012-13-1-r3 PMC3334584

[95] Moriyama Y. , Koshiba-Takeuchi K. 2018, Significance of whole-genome duplications on the emergence of evolutionary novelties, Brief. Funct. Genom., 17, 329–38. 10.1093/bfgp/ely007 29579140

[96] Wu Z. , Liu H., Zhan W., et al 2021, The chromosome-scale reference genome of safflower (*Carthamus tinctorius*) provides insights into linoleic acid and flavonoid biosynthesis, Plant Biotechnol. J., 19, 1725–42. 33768699 10.1111/pbi.13586 PMC8428823

[97] Wang Y. , Ficklin S.P., Wang X., Feltus F.A., Paterson A.H. 2016, Large-scale gene relocations following an ancient genome triplication associated with the diversification of core eudicots, PLoS One, 11, e0155637. 27195960 10.1371/journal.pone.0155637 PMC4873151

[98] Lee H.Y. , Mang H., Choi E., et al 2021, Genome-wide functional analysis of hot pepper immune receptors reveals an autonomous NLR clade in seed plants, New Phytol., 229, 532–47. 32810286 10.1111/nph.16878 PMC7756659

[99] Zhang Y.M. , Chen M., Sun L., et al 2020, Genome-wide identification and evolutionary analysis of NBS-LRR genes from *Dioscorea rotundata*, Front. Genet., 11, 484. 32457809 10.3389/fgene.2020.00484 PMC7224235

[100] Ma Y. , Chhapekar S.S., Lu L., et al 2021, Genome-wide identification and characterization of NBS-encoding genes in *Raphanus sativus* L. and their roles related to *Fusarium oxysporum* resistance, BMC Plant Biol., 21, 47. 33461498 10.1186/s12870-020-02803-8 PMC7814608

[101] Baggs E.L. , Monroe J.G., Thanki A.S., et al 2020, Convergent loss of an EDS1/PAD4 signaling pathway in several plant lineages reveals coevolved components of plant immunity and drought response, Plant Cell, 32, 2158–77. 32409319 10.1105/tpc.19.00903 PMC7346574

