# Supplementary material for: Chromosome-level genome assembly of the aquatic plant Nymphoides indica reveals transposable element bursts and NBS-LRR gene family expansion shedding light on its invasiveness
Source: DNA Res. 2022 Jun 25;29(4):dsac022. doi: 10.1093/dnares/dsac022 (PMC9267246; doi:10.1093/dnares/dsac022)
Supplement: dsac022_Supplementary_Data [file dsac022_supplementary_data.docx]

**Supplemental Information for**

**Chromosome-level genome assembly of the aquatic plant *Nymphoides indica* reveals transposable element bursts and NBS-LRR gene family expansion shedding light on its invasiveness**

**Supplementary Table 1 Download links for genomic profiles used in this study.**

| **Species** | **links** |
| --- | --- |
| *A. thaliana* | https://data.jgi.doe.gov/refine-download/phytozome?genome_id=167 |
| *A. trichopoda* | https://data.jgi.doe.gov/refine-download/phytozome?genome_id=291 |
| *V. vinifera* | https://data.jgi.doe.gov/refine-download/phytozome?genome_id=457 |
| *H. annuus* | https://data.jgi.doe.gov/refine-download/phytozome?genome_id=494 |
| *C. cardunculus* | www.artichokegenome.unito.it |
| *C. canephora* | https://www.ncbi.nlm.nih.gov/assembly/GCA_900059795.1 |
| *O. sativa* | http:www.insect-genome.com/chilo/ |
| *E. ferox* | https://genomevolution.org/CoGe/GenomeInfo.pl?gid=56574 |
| *N. colorata* | https://ngdc.cncb.ac.cn/search/?dbId=gwh&q=GWHAAYW00000000&page=1 |
| *N. nucifera* | http://nelumbo.biocloud.net/nelumbo/download/download |
| *Z. marina* | https://www.ncbi.nlm.nih.gov/nuccore/JABCRI000000000.1 |
| *S. polyrhiza* | https://data.jgi.doe.gov/refine-download/phytozome?genome_id=290 |
| *T. sinense* | https://www.ncbi.nlm.nih.gov/data-hub/taxonomy/13715/?utm_source=datasets&utm_medium=referral&utm_campaign=genome-table&utm_term=Tetracentron%20sinense |

**Supplementary Table 2** **Sequencing information of *N. indica*.**

| **Type** | **Design description** | **Platform** | **Read number** | **Total data (G)** | **Read length (bp)** | **Sequence coverage (×)** |
| --- | --- | --- | --- | --- | --- | --- |
| Illumina short reads | leaf | Hiseq X | 242,949,079 | 72.88 | 150 | 117 |
| PacBio long reads | leaf | PacBio Sequel | 6,712,790 | 142 | 21,150 | 227 |
| Hi-C reads | leaf | HiSeq X | 1,258,686,972 | 93.59 | 150 | 150 |

**Supplementary Table 3 Overview of chromosome length and scaffold numbers on each chromosome of *N. indica* assembly**

| **Chr_ID** | **Number of Contigs** | **Chr_Length (bp)** |
| --- | --- | --- |
| Chr1 | 11 | 66,869,476 |
| Chr2 | 9 | 55,962,388 |
| Chr3 | 20 | 52,213,865 |
| Chr4 | 7 | 52,206,535 |
| Chr5 | 12 | 49,182,702 |
| Chr6 | 22 | 46,488,395 |
| Chr7 | 13 | 46,013,668 |
| Chr8 | 12 | 45,644,229 |
| Chr9 | 18 | 40,698,313 |
| Total | 124 | 455,279,571 |

**Supplementary Table 4 BUSCO analysis of genome assembly of *N. indica***

| **Description** | **Number** | **Percentage (%)** |
| --- | --- | --- |
| Complete BUSCOs (C) | 1,530 | 94.80 |
| Complete and single-copy BUSCOs (S) | 1,495 | 92.60 |
| Complete and duplicated BUSCOs (D) | 35 | 2.20 |
| Fragmented BUSCOs (F) | 14 | 0.90 |
| Missing BUSCOs (M) | 70 | 4.30 |
| Total BUSCO groups searched | 1,614 | 100.00 |

**Supplementary Table 5 Protein-coding gene prediction.**

| **Method** | **Software** | **Gene number** |
| --- | --- | --- |
| Ab initio | Augustus | 51,860 |
| Homology-based | Genewise | 60,785 |
| RNAseq | PASA | 32,449 |
|  | TransDecoder | 19,151 |
| Integration and update | EVM (integration)+PASA (update) | 29,938 |

**Supplementary Table 6 Statistics on the annotation of the *N. indica* genome.**

|  | | **Number** | **Percent (%)** |
| --- | --- | --- | --- |
| Total | | 29,983 | 100.00 |
| Annotated | InterPro | 23,787 | 79.32 |
|  | eggNOG | 24,121 | 80.44 |
|  | Swissprot | 16,923 | 56.44 |
|  | NR | 24,871 | 83.00 |

**Supplementary Table 7 Statistics transcription factors of the *N. indica* genome**

| **Classify** | **Number** |
| --- | --- |
| AP2 family protein | 15 |
| ARF family protein | 14 |
| ARR-B family protein | 11 |
| B3 family protein | 76 |
| BBR-BPC family protein | 4 |
| BES1 family protein | 8 |
| bHLH family protein | 104 |
| bZIP family protein | 43 |
| C2H2 family protein | 75 |
| C3H family protein | 38 |
| CAMAT family protein | 4 |
| CO-like family protein | 8 |
| CPP family protein | 4 |
| DBB family protein | 6 |
| Dof family protein | 19 |
| E2F transcription factor | 6 |
| EIL family protein | 6 |
| ERF family protein | 98 |
| FAR1 family protein | 32 |
| G2-like family protein | 32 |
| GATA transcription factor | 20 |
| GRAS family protein | 48 |
| GRF family protein | 8 |
| HB-other family protein | 8 |
| HB-PHD family protein | 2 |
| HD-ZIP family protein | 31 |
| HRT-like family protein | 3 |
| HSF family protein | 17 |
| LFY family protein | 1 |
| LBD family protein | 32 |
| LSD family protein | 3 |
| M-type_MADS family protein | 25 |
| MIKC_MADS family protein | 24 |
| MYB family protein | 98 |
| MYB_related family protein | 49 |
| NAC family protein | 87 |
| NF-X1 family protein | 3 |
| NF-YA family protein | 6 |
| NF-YB family protein | 17 |
| NF-YC family protein | 5 |
| Nin-like | 14 |
| RAV family protein | 2 |
| S1FA-like DNA-binding protein | 1 |
| SAP family protein | 1 |
| SBP family protein | 16 |
| SRS family protein | 5 |
| STAT | 1 |
| TALE | 14 |
| TCP family protein | 15 |
| Trihelix | 38 |
| VOZ | 1 |
| WOX | 9 |
| WRKY family protein | 44 |
| YABBY family protein | 5 |
| ZF-HD family protein | 7 |

**Supplementary Table 8 TE annotation of *N. indica* genome**

| **Class** | **Count** | **Length (bp)** | **Percentage of the genome (%)** |
| --- | --- | --- | --- |
| **LTR** |  |  |  |
| Copia | 63,633 | 48,505,415 | 9.26 |
| Gypsy | 64,843 | 87,894,887 | 16.78 |
| unknown | 79,941 | 41,684,903 | 7.96 |
| **TIR** |  |  |  |
| CACTA | 36,993 | 12,335,319 | 2.35 |
| Mutator | 126,064 | 29,753,039 | 5.68 |
| PIF_Harbinger | 38,423 | 7,960,232 | 1.52 |
| Tc1_Mariner | 14,579 | 2,713,035 | 0.52 |
| hAT | 45,134 | 13,082,095 | 2.50 |
| **nonTIR** |  |  |  |
| helitron | 119,732 | 30,043,800 | 5.73 |
| **Total** | 589,342 | 273,972,725 | 52.29 |

**Supplementary Table 9 Summary of gene family clustering**

| **Species** | **Total genes** | **Number of genes in orthogroups** | **Number of species-specific orthogroups** | **Number of genes in species-specific orthogroups** |
| --- | --- | --- | --- | --- |
| *A. thaliana* | 27,416 | 24,921 | 706 | 3,465 |
| *A. trichopoda* | 26,846 | 22,318 | 831 | 4,236 |
| *V. vinifera* | 31,845 | 27,529 | 727 | 2,615 |
| *H. annuus* | 52,243 | 44,711 | 1,847 | 7,458 |
| *C. dunculus* | 28,632 | 26,951 | 583 | 2,630 |
| *C. canephora* | 25,574 | 23,401 | 428 | 2,099 |
| *O. sativa* | 42,189 | 33,514 | 2,155 | 11,425 |
| *E. ferox* | 40,049 | 34,801 | 830 | 2,630 |
| *N. colorata* | 31,475 | 27,166 | 770 | 4,419 |
| *N. nucifera* | 32,124 | 29,434 | 596 | 2,277 |
| *Z. marina* | 20,450 | 18,662 | 415 | 2,059 |
| *S. polyrhiza,* | 19,623 | 18,108 | 299 | 1,520 |
| *T. sinense* | 32,687 | 30,643 | 565 | 2,078 |
| *N. indica* | 29,983 | 27,286 | 926 | 4,249 |

**Supplementary Table 10 GO functional enrichment analysis of *N. indica*-specific gene families**

| **ID** | **Description** | **count** | ***P*-value** |
| --- | --- | --- | --- |
| GO:0000741 | karyogamy | 21 | 3.96E-15 |
| GO:0010197 | polar nucleus fusion | 21 | 3.96E-15 |
| GO:0009559 | embryo sac central cell differentiation | 21 | 4.71E-14 |
| GO:0009561 | megagametogenesis | 27 | 5.89E-13 |
| GO:0035694 | mitochondrial protein catabolic process | 11 | 6.05E-13 |
| GO:0004869 | cysteine-type endopeptidase inhibitor activity | 16 | 1.19E-12 |
| GO:0004866 | endopeptidase inhibitor activity | 16 | 3.93E-12 |
| GO:0030414 | peptidase inhibitor activity | 16 | 3.93E-12 |
| GO:0010466 | negative regulation of peptidase activity | 16 | 8.69E-12 |
| GO:0010951 | negative regulation of endopeptidase activity | 16 | 8.69E-12 |
| GO:0006997 | nucleus organization | 23 | 8.92E-12 |
| GO:0061135 | endopeptidase regulator activity | 16 | 1.97E-11 |
| GO:0061134 | peptidase regulator activity | 16 | 3.24E-11 |
| GO:0004867 | serine-type endopeptidase inhibitor activity | 10 | 4.98E-11 |
| GO:0046658 | anchored component of plasma membrane | 27 | 7.67E-11 |
| GO:0031929 | TOR signaling | 12 | 1.76E-10 |
| GO:0052548 | regulation of endopeptidase activity | 16 | 2.23E-10 |
| GO:0052547 | regulation of peptidase activity | 16 | 3.33E-10 |
| GO:0031225 | anchored component of membrane | 28 | 7.03E-10 |
| GO:0001871 | pattern binding | 19 | 1.78E-09 |

**Supplementary Table 11 KEGG functional enrichment analysis of *N. indica*-specific gene families**

| **ID** | **Descrption** | **count** | ***P*-value** |
| --- | --- | --- | --- |
| ko01040 | Biosynthesis of unsaturated fatty acids | 20 | 7.12E-10 |
| ko01212 | Fatty acid metabolism | 20 | 1.40E-06 |
| ko00750 | Vitamin B6 metabolism | 7 | 0.000125 |
| ko03060 | Protein export | 11 | 0.000887 |
| ko00901 | Indole alkaloid biosynthesis | 3 | 0.001556 |
| ko00500 | Starch and sucrose metabolism | 21 | 0.002314 |
| ko00260 | Glycine, serine and threonine metabolism | 15 | 0.002434 |
| ko00906 | Carotenoid biosynthesis | 7 | 0.002648 |
| ko00730 | Thiamine metabolism | 6 | 0.00446 |
| ko01100 | Metabolic pathways | 174 | 0.004753 |
| ko00073 | Cutin, suberine and wax biosynthesis | 6 | 0.010004 |
| ko00052 | Galactose metabolism | 8 | 0.018522 |
| ko04141 | Protein processing in endoplasmic reticulum | 20 | 0.020123 |
| ko00190 | Oxidative phosphorylation | 13 | 0.028985 |
| ko00592 | alpha-Linolenic acid metabolism | 8 | 0.033348 |
| ko00909 | Sesquiterpenoid and triterpenoid biosynthesis | 5 | 0.033636 |

**Supplementary Table 12 GO functional enrichment analysis of expanded gene families in *N. indica***

| **ID** | **Description** | **count** | ***P*-value** |
| --- | --- | --- | --- |
| GO:0009505 | plant-type cell wall | 178 | 1.85E-20 |
| GO:0016788 | hydrolase activity, acting on ester bonds | 412 | 1.63E-20 |
| GO:0000943 | retrotransposon nucleocapsid | 49 | 1.48E-18 |
| GO:0032196 | transposition | 55 | 1.45E-19 |
| GO:0003964 | RNA-directed DNA polymerase activity | 49 | 4.89E-18 |
| GO:0003824 | catalytic activity | 1,640 | 3.14E-17 |
| GO:0032197 | transposition, RNA-mediated | 49 | 2.19E-17 |
| GO:0052689 | carboxylic ester hydrolase activity | 83 | 7.18E-15 |
| GO:0004518 | nuclease activity | 262 | 1.22E-14 |
| GO:0004016 | adenylate cyclase activity | 25 | 1.13E-13 |
| GO:0016787 | hydrolase activity | 718 | 2.36E-13 |
| GO:0090615 | mitochondrial mRNA processing | 31 | 3.41E-14 |
| GO:0003887 | DNA-directed DNA polymerase activity | 49 | 3.92E-13 |
| GO:0090353 | polygalacturonase inhibitor activity | 24 | 4.04E-13 |
| GO:0034061 | DNA polymerase activity | 49 | 1.93E-12 |
| GO:0030599 | pectinesterase activity | 43 | 9.42E-12 |
| GO:0016491 | oxidoreductase activity | 308 | 1.15E-11 |
| GO:0000963 | mitochondrial RNA processing | 37 | 1.64E-12 |
| GO:0006278 | RNA-dependent DNA replication | 54 | 3.10E-12 |
| GO:0005618 | cell wall | 256 | 1.28E-10 |

**Supplementary Table 13 KEGG functional enrichment analysis of expanded gene families in *N. indica***

| **ID** | **Descrption** | **count** | ***P*-value** |
| --- | --- | --- | --- |
| ko00940 | Phenylpropanoid biosynthesis | 147 | 2.80E-26 |
| ko00905 | Brassinosteroid biosynthesis | 23 | 7.69E-10 |
| ko01110 | Biosynthesis of secondary metabolites | 482 | 1.08E-08 |
| ko00350 | Tyrosine metabolism | 38 | 3.26E-08 |
| ko00040 | Pentose and glucuronate interconversions | 75 | 4.07E-08 |
| ko00514 | Other types of O-glycan biosynthesis | 21 | 1.82E-06 |
| ko00904 | Diterpenoid biosynthesis | 26 | 7.15E-06 |
| ko00460 | Cyanoamino acid metabolism | 35 | 7.88E-06 |
| ko04626 | Plant-pathogen interaction | 104 | 1.18E-05 |
| ko01100 | Metabolic pathways | 825 | 4.48E-05 |
| ko01040 | Biosynthesis of unsaturated fatty acids | 35 | 9.19E-05 |
| ko00591 | Linoleic acid metabolism | 15 | 9.36E-05 |
| ko00966 | Glucosinolate biosynthesis | 16 | 0.000327 |
| ko00970 | Aminoacyl-tRNA biosynthesis | 34 | 0.001113 |
| ko00944 | Flavone and flavonol biosynthesis | 5 | 0.001662 |
| ko00071 | Fatty acid degradation | 27 | 0.00235 |
| ko00941 | Flavonoid biosynthesis | 23 | 0.003248 |
| ko00950 | Isoquinoline alkaloid biosynthesis | 15 | 0.003499 |
| ko00360 | Phenylalanine metabolism | 19 | 0.00434 |
| ko00260 | Glycine, serine and threonine metabolism | 47 | 0.004345 |

**Supplementary Table 14 GO functional enrichment analysis of rapidly expanded gene families in *N. indica***

| **ID** | **Descrption** | **count** | ***P*-value** |
| --- | --- | --- | --- |
| GO:0003964 | RNA-directed DNA polymerase activity | 45 | 9.84E-63 |
| GO:0000943 | retrotransposon nucleocapsid | 45 | 1.88E-61 |
| GO:0003887 | DNA-directed DNA polymerase activity | 45 | 1.88E-57 |
| GO:0034061 | DNA polymerase activity | 45 | 1.21E-56 |
| GO:0032197 | transposition, RNA-mediated | 45 | 9.88E-57 |
| GO:0032196 | transposition | 45 | 4.19E-53 |
| GO:0006278 | RNA-dependent DNA replication | 45 | 3.01E-47 |
| GO:0004540 | ribonuclease activity | 45 | 6.14E-42 |
| GO:0004857 | enzyme inhibitor activity | 46 | 2.63E-40 |
| GO:0090353 | polygalacturonase inhibitor activity | 24 | 2.22E-39 |
| GO:0071897 | DNA biosynthetic process | 45 | 8.97E-39 |
| GO:0016779 | nucleotidyltransferase activity | 45 | 1.14E-37 |
| GO:0008233 | peptidase activity | 59 | 6.41E-37 |
| GO:0009505 | plant-type cell wall | 59 | 5.25E-33 |
| GO:0090501 | RNA phosphodiester bond hydrolysis | 45 | 2.45E-31 |
| GO:0030234 | enzyme regulator activity | 46 | 8.44E-29 |
| GO:0043086 | negative regulation of catalytic activity | 46 | 2.92E-28 |
| GO:0071944 | cell periphery | 154 | 2.04E-27 |
| GO:0044092 | negative regulation of molecular function | 46 | 3.69E-27 |
| GO:0098772 | molecular function regulator | 46 | 4.02E-26 |
| GO:0006260 | DNA replication | 46 | 6.00E-26 |
| GO:0001666 | response to hypoxia | 34 | 2.63E-25 |
| GO:0036293 | response to decreased oxygen levels | 34 | 1.54E-23 |
| GO:0070482 | response to oxygen levels | 34 | 2.06E-23 |

**Supplementary Table 15 KEGG functional enrichment analysis of rapidly expanded gene families in *N. indica.***

| **ID** | **Descrption** | **count** | ***P*-value** |
| --- | --- | --- | --- |
| ko00940 | Phenylpropanoid biosynthesis | 49 | 7.34E-21 |
| ko04626 | Plant-pathogen interaction | 50 | 7.34E-21 |
| ko00040 | Pentose and glucuronate interconversions | 34 | 6.38E-16 |
| ko00520 | Amino sugar and nucleotide sugar metabolism | 30 | 6.02E-12 |
| ko01040 | Biosynthesis of unsaturated fatty acids | 19 | 7.55E-11 |
| ko01212 | Fatty acid metabolism | 19 | 1.29E-07 |
| ko04130 | SNARE interactions in vesicular transport | 11 | 2.48E-06 |
| ko01100 | Metabolic pathways | 143 | 4.86E-06 |
| ko03022 | Basal transcription factors | 12 | 1.12E-05 |

**Supplementary Table 16 GO functional enrichment analysis of WGT-related genes in *N. indica***

| **ID** | **Descrption** | **count** | ***P*-value** |
| --- | --- | --- | --- |
| GO:0001071 | nucleic acid binding transcription factor activity | 184 | 1.16E-18 |
| GO:0003700 | transcription factor activity, sequence-specific DNA binding | 184 | 1.16E-18 |
| GO:0034654 | nucleobase-containing compound biosynthetic process | 278 | 4.06E-14 |
| GO:1903506 | regulation of nucleic acid-templated transcription | 234 | 7.95E-14 |
| GO:2001141 | regulation of RNA biosynthetic process | 234 | 7.95E-14 |
| GO:0006355 | regulation of transcription, DNA-templated | 231 | 7.99E-14 |
| GO:0097659 | nucleic acid-templated transcription | 238 | 1.44E-12 |
| GO:0006351 | transcription, DNA-templated | 235 | 1.73E-12 |
| GO:0032774 | RNA biosynthetic process | 238 | 2.82E-12 |
| GO:0051252 | regulation of RNA metabolic process | 237 | 9.63E-12 |
| GO:1902495 | transmembrane transporter complex | 11 | 1.47E-11 |
| GO:0005886 | plasma membrane | 332 | 1.86E-11 |
| GO:2000112 | regulation of cellular macromolecule biosynthetic process | 246 | 1.36E-11 |
| GO:0010556 | regulation of macromolecule biosynthetic process | 251 | 1.58E-11 |
| GO:0018130 | heterocycle biosynthetic process | 291 | 2.21E-11 |
| GO:0019438 | aromatic compound biosynthetic process | 298 | 4.24E-11 |
| GO:0031326 | regulation of cellular biosynthetic process | 255 | 1.15E-10 |
| GO:0071944 | cell periphery | 385 | 1.88E-10 |
| GO:0019219 | regulation of nucleobase-containing compound metabolic process | 241 | 3.56E-10 |
| GO:0051171 | regulation of nitrogen compound metabolic process | 284 | 6.12E-10 |

**Supplementary Table 17 KEGG functional enrichment analysis of WGT-related genes in *N. indica***

| **ID** | **Descrption** | **count** | ***P*-value** |
| --- | --- | --- | --- |
| ko04120 | Ubiquitin mediated proteolysis | 33 | 2.22E-05 |
| ko00010 | Glycolysis / Gluconeogenesis | 25 | 0.001637 |
| ko00051 | Fructose and mannose metabolism | 13 | 0.001988 |
| ko00710 | Carbon fixation in photosynthetic organisms | 14 | 0.003437 |
| ko04130 | SNARE interactions in vesicular transport | 10 | 0.00857 |
| ko00561 | Glycerolipid metabolism | 16 | 0.009279 |
| ko00520 | Amino sugar and nucleotide sugar metabolism | 26 | 0.009331 |
| ko00564 | Glycerophospholipid metabolism | 18 | 0.012409 |
| ko00310 | Lysine degradation | 10 | 0.020867 |
| ko04016 | MAPK signaling pathway - plant | 20 | 0.022884 |
| ko04070 | Phosphatidylinositol signaling system | 13 | 0.028449 |
| ko03022 | Basal transcription factors | 11 | 0.036025 |

**Supplementary Table 18 NBS-LRR genes and their classification in *N. indica* genome**

| **Predicted domains** | **Number** |
| --- | --- |
| CNL Type | 68 |
| TNL Type | 50 |
| RNL Type | 18 |
| ANL Type | 24 |
| Total | 160 |

**Supplementary Table 19 The gene expression FPKM of NBS-LRR genes in *N. indica* genome. The gray indicated the 44 NBS-LRR genes that exhibited higher expression levels of leaf than other tissues)**

| Gene ID | flower | root | stem | leaf |
| --- | --- | --- | --- | --- |
| NinS00354 | 0.386013 | 5.074341 | 0.037055 | 0.486456 |
| NinS00375 | 0.536195 | 0.011759 | 16.96064 | 0 |
| NinS00378 | 0 | 0.071677 | 1.612685 | 0 |
| NinS00541 | 9.750347 | 2.987892 | 54.15664 | 0 |
| NinS00616 | 0 | 36.50164 | 53.12823 | 15.37485 |
| NinS00846 | 0 | 0.062392 | 63.48129 | 10.25564 |
| NinS00848 | 0.954953 | 1.111284 | 11.30867 | 20.45981 |
| NinS00855 | 54.81599 | 1.187655 | 26.65511 | 6.300179 |
| NinS00856 | 22.11144 | 0.327514 | 0.291833 | 0.314696 |
| NinS00879 | 0 | 0.528102 | 0 | 0 |
| NinS00881 | 1.681378 | 0 | 1.03195 | 0 |
| NinS04079 | 32.51055 | 0.271141 | 0.136842 | 0 |
| NinS05729 | 4.573647 | 0.011237 | 0 | 60.508 |
| NinS05732 | 39.23139 | 0 | 15.92396 | 1.490218 |
| NinS06159 | 19.19796 | 3.263948 | 5.955772 | 28.74469 |
| NinS06896 | 41.02742 | 3.803536 | 0 | 49.59419 |
| NinS06889 | 7.281217 | 34.34699 | 2.501768 | 288.1582 |
| NinS06891 | 0.047407 | 14.91435 | 2.681024 | 17.3878 |
| NinS07255 | 0.280077 | 27.8889 | 3.505637 | 0 |
| NinS07256 | 4.081333 | 90.80121 | 32.06012 | 85.15044 |
| NinS07260 | 10.4869 | 0.210378 | 31.52274 | 930.9976 |
| NinS09643 | 16.8417 | 1.476813 | 3.11851 | 13.79391 |
| NinS09998 | 0 | 14.05959 | 5.714299 | 38.8991 |
| NinS10872 | 2.083596 | 80.66678 | 17.88364 | 6.663946 |
| NinS10873 | 18.17893 | 15.35244 | 1.280387 | 3.479491 |
| NinS10874 | 10.60371 | 3.150603 | 6.342836 | 0.229174 |
| NinS10875 | 71.2577 | 32.93395 | 81.87499 | 3.207705 |
| NinS10876 | 1.741615 | 0.579652 | 44.49097 | 0.104036 |
| NinS12702 | 77.55 | 57.83621 | 77.39957 | 0.789271 |
| NinS12738 | 350.1316 | 0.217188 | 9.682303 | 17.5267 |
| NinS12739 | 0 | 11.22959 | 81.59267 | 0 |
| NinS14012 | 0.030555 | 7.371481 | 20.28654 | 11.36212 |
| NinS14962 | 0.067712 | 0 | 0.56829 | 0.429547 |
| NinS16052 | 12.62445 | 5.550861 | 0.452772 | 15.75814 |
| NinS16054 | 43.77598 | 110.3427 | 6.193798 | 139.0937 |
| NinS16175 | 17.29312 | 0 | 0.139558 | 26.02461 |
| NinS16174 | 1.31951 | 98.48863 | 1.439048 | 3.1514 |
| NinS16180 | 19.54419 | 1.077695 | 0.534206 | 2.140121 |
| NinS16183 | 7.64326 | 8.971231 | 0.18015 | 7.210871 |
| NinS16184 | 7.760238 | 31.89443 | 40.89097 | 0.634528 |
| NinS16186 | 22.26882 | 11.78022 | 0 | 0 |
| NinS16195 | 32.28489 | 0.020196 | 0 | 28.51761 |
| NinS16196 | 16.05552 | 1.898236 | 0 | 39.80299 |
| NinS16199 | 50.24643 | 0.044934 | 0 | 0 |
| NinS16203 | 0.055368 | 37.81172 | 0 | 2.950412 |
| NinS16205 | 7.879699 | 0 | 0.990191 | 0.466274 |
| NinS16206 | 39.67445 | 0 | 6.723527 | 11.99128 |
| NinS16210 | 26.85975 | 8.486798 | 32.59328 | 0.327826 |
| NinS16214 | 17.5203 | 22.02029 | 8.397717 | 0 |
| NinS16219 | 27.88138 | 34.22829 | 0 | 1.286266 |
| NinS16221 | 37.07861 | 25.05051 | 0 | 88.06159 |
| NinS16263 | 53.26317 | 1.215138 | 6.67021 | 0.175651 |
| NinS16609 | 12.49517 | 1.085141 | 0.101178 | 14.53952 |
| NinS16710 | 73.59028 | 0 | 0.089866 | 0 |
| NinS17360 | 11.40845 | 0.278408 | 53.31636 | 0.108127 |
| NinS17361 | 0.366423 | 3.020074 | 1.914733 | 2.872609 |
| NinS17368 | 0.114632 | 200.7808 | 2.857793 | 0.057737 |
| NinS17372 | 0.495354 | 0.506674 | 12.90716 | 0.344932 |
| NinS17373 | 0.31684 | 0.153419 | 0.382033 | 2.254868 |
| NinS17395 | 0 | 2.237833 | 2.110521 | 17.0127 |
| NinS17396 | 0.217181 | 1.523559 | 1.617627 | 6.223765 |
| NinS17400 | 0.418975 | 0.158842 | 3.271486 | 9.111834 |
| NinS17561 | 0.008257 | 2.228582 | 0 | 60.25866 |
| NinS17562 | 1.250883 | 5.34769 | 0 | 19.32979 |
| NinS17950 | 0.509529 | 2.947124 | 0 | 2.043864 |
| NinS18301 | 0.461858 | 1.134955 | 7.397188 | 0 |
| NinS18567 | 4.41112 | 1.705753 | 17.6809 | 18.80422 |
| NinS18568 | 18.45567 | 0.155907 | 5.445922 | 0.044373 |
| NinS18574 | 18.5162 | 24.92658 | 23.67561 | 1.766963 |
| NinS18553 | 20.97705 | 4.584527 | 4.983698 | 11.82245 |
| NinS18587 | 0 | 10.44664 | 15.50684 | 17.62512 |
| NinS18600 | 6.924167 | 27.6552 | 28.31215 | 22.31229 |
| NinS18602 | 0 | 13.49302 | 0 | 0.062852 |
| NinS18606 | 0.07325 | 0.076256 | 2.388983 | 50.79124 |
| NinS19045 | 0.343356 | 2.218092 | 0.493817 | 0 |
| NinS20584 | 9.515684 | 3.422856 | 2.978391 | 7.820582 |
| NinS20593 | 308.96 | 0 | 1.885344 | 0 |
| NinS20601 | 4.490196 | 9.882837 | 4.765368 | 0 |
| NinS20602 | 2.337627 | 637.5892 | 0.069429 | 6.150767 |
| NinS20608 | 39.09841 | 139.7238 | 0.165782 | 0.427116 |
| NinS20664 | 69.78518 | 8.63101 | 0.111915 | 25.88148 |
| NinS20727 | 1.053193 | 139.0272 | 0.209623 | 70.46503 |
| NinS20733 | 7.442104 | 21.44625 | 0 | 4.19542 |
| NinS20735 | 22.94329 | 25.7339 | 2.293703 | 8.038808 |
| NinS20736 | 0.040304 | 7.20189 | 0.563464 | 13.36703 |
| NinS20738 | 18.83754 | 0 | 1.22174 | 8.676695 |
| NinS20740 | 18.29256 | 113.1522 | 0.280167 | 0.515387 |
| NinS20741 | 46.16489 | 0 | 3.732415 | 1397.604 |
| NinS20780 | 0.617179 | 0.060085 | 6.545429 | 0.063965 |
| NinS20971 | 6.318008 | 0.241981 | 0.27093 | 354.977 |
| NinS20970 | 0.430637 | 0 | 0.278446 | 4.580464 |
| NinS20972 | 19.27224 | 1.290825 | 1.587509 | 54.33166 |
| NinS20976 | 4.253975 | 18.80928 | 1.325397 | 21.83479 |
| NinS20999 | 0 | 0 | 0.671301 | 1.71363 |
| NinS21059 | 5.075834 | 85.15537 | 9.528998 | 44.38313 |
| NinS21061 | 84.34592 | 61.64568 | 0.119094 | 24.35535 |
| NinS21066 | 0.179312 | 0 | 0 | 7.535037 |
| NinS21068 | 57.76659 | 0.044 | 13.60917 | 0 |
| NinS21071 | 0 | 21.65224 | 8.732793 | 39.74337 |
| NinS21078 | 0.086484 | 0 | 14.3264 | 41.80303 |
| NinS21080 | 0 | 0.165685 | 6.05816 | 1.171261 |
| NinS21082 | 0.689762 | 0 | 15.44675 | 13.86358 |
| NinS21083 | 0 | 0 | 7.743166 | 3.143684 |
| NinS21084 | 6.083574 | 0 | 46.96719 | 0.157486 |
| NinS21522 | 73.88158 | 49.17486 | 2.101552 | 1.214886 |
| NinS22531 | 2.361109 | 0 | 0 | 2.951001 |
| NinS22537 | 31.31421 | 26.51572 | 13.22112 | 10.15653 |
| NinS22540 | 11.19622 | 0.613931 | 4.387978 | 0 |
| NinS22541 | 5.366457 | 0.165237 | 10.99281 | 0.021911 |
| NinS22544 | 56.15848 | 2.400877 | 0 | 1.279657 |
| NinS22546 | 1.384821 | 91.79862 | 60.31499 | 103.5268 |
| NinS22547 | 6.713036 | 2.00281 | 0 | 2.827787 |
| NinS22550 | 11.84694 | 53.57884 | 14.13612 | 21.4475 |
| NinS22551 | 0.434254 | 0 | 7.264308 | 3.384113 |
| NinS22553 | 6.947548 | 1.117902 | 0.19587 | 6.08385 |
| NinS22637 | 18.37991 | 0.946272 | 4.285987 | 1.294131 |
| NinS22876 | 24.61217 | 13.31234 | 10.14562 | 0.318298 |
| NinS23717 | 13.53785 | 22.66079 | 35.35272 | 9.395509 |
| NinS24550 | 17.40386 | 18.95375 | 0.471026 | 16.0295 |
| NinS24644 | 3.435562 | 146.532 | 18.75072 | 0.135384 |
| NinS24641 | 97.74444 | 6.33707 | 0.312429 | 29.34629 |
| NinS24599 | 17.38777 | 21.82177 | 15.67516 | 45.14171 |
| NinS24605 | 20.74116 | 3.672757 | 1.028782 | 7.675673 |
| NinS24609 | 11.51963 | 173.1205 | 8.681867 | 10.47718 |
| NinS24620 | 0 | 9.220436 | 12.43344 | 17.26779 |
| NinS24623 | 12.9308 | 0 | 21.67334 | 34.67267 |
| NinS24626 | 34.21495 | 8.261875 | 0 | 12.35428 |
| NinS24628 | 17.40225 | 1.670599 | 24.12151 | 3.077022 |
| NinS24633 | 0.080302 | 4.757931 | 0.208337 | 21.42084 |
| NinS24662 | 21.97321 | 0.042471 | 37.21349 | 4.619785 |
| NinS24663 | 80.52683 | 2.528491 | 0.012144 | 935.4623 |
| NinS24668 | 25.33451 | 45.59998 | 5.055652 | 58.27602 |
| NinS24650 | 10.25173 | 11.85475 | 2.170546 | 0.569558 |
| NinS24653 | 3.009559 | 2.051595 | 0.19874 | 2.881165 |
| NinS24657 | 2.007462 | 0 | 51.24762 | 0.698512 |
| NinS24672 | 80.86475 | 7.271531 | 6.576006 | 10.24978 |
| NinS25287 | 21.12551 | 221.5087 | 21.87395 | 0.060564 |
| NinS27651 | 1.534797 | 0 | 0 | 0.428584 |
| NinS28600 | 0.315708 | 8.776223 | 7.494347 | 9.294777 |
| NinS29005 | 19.88662 | 0.040909 | 0.019385 | 1.353975 |
| NinS29009 | 1.710103 | 1.368981 | 0 | 0.199774 |
| NinS01417 | 1.466586 | 13.4008 | 0 | 0 |
| NinS01419 | 0.438703 | 1.557455 | 0 | 23.76162 |
| NinS01729 | 0.03618 | 0.441495 | 0 | 0 |
| NinS01418 | 0.324928 | 7.414187 | 0 | 8.426146 |
| NinS00363 | 47.83068 | 0.244427 | 0 | 0.821211 |
| NinS01725 | 5.421993 | 0.28398 | 0 | 0 |
| NinS06900 | 0 | 0 | 0 | 0 |
| NinS17363 | 0 | 0 | 0 | 0 |
| NinS16618 | 0 | 0 | 0 | 0 |
| NinS17370 | 0 | 0 | 0 | 0 |
| NinS17364 | 0 | 0 | 0 | 0 |
| NinS17371 | 0 | 0 | 0 | 0 |
| NinS18560 | 0 | 0 | 0 | 0 |
| NinS18582 | 0 | 0 | 0 | 0 |
| NinS18549 | 0 | 0 | 0 | 0 |
| NinS20771 | 0 | 0 | 0 | 0 |
| NinS18558 | 0 | 0 | 0 | 0 |
| NinS24665 | 0 | 0 | 0 | 0 |
| NinS24666 | 0 | 0 | 0 | 0 |

**Supplementary Table 20 Homologs of known immunity genes in *N. indica* from reciprocal BLAST searches against *A. thaliana*. Gene names given as in *A. thaliana*.**

| **Gene ID** | **Gene name** | **Homologue in *N. indica*** |
| --- | --- | --- |
| AT5G13160 | PBS1 | NinS00087 |
| AT3G25070 | RIN4 | NinS06404, NinS18302, NinS21151, NinS28937 |
| AT1G18870 | ICS2 | NinS03770 |
| AT2G43820 | SGT1 | NinS21394, NinS23195 |
| AT1G64280 | NPR1 | NinS12298 |
| AT4G26120 | NPR2 | None |
| AT3G48090 | EDS1 | NinS03840, NinS03841, NinS03842, NinS03843 |
| AT3G52430 | PAD4 | NinS07938 |
| AT5G14930 | SAG101 | NinS10357, NinS10358, NinS10359, NinS10360 |
| AT4G33300 | ADR1-L1 (NRG1) | NinS04079 |
| AT4G39030 | SID1 | None |
| AT1G74710 | SID2/ICS1 | None |
| AT1G19250 | FMO1 | NinS01090, NinS01091, NinS12169, NinS24709, NinS24710 |
| AT2G13810 | ALD1 | NinS08981 |
| AT1G02170 | MC1 | NinS16645, NinS21909, NinS21911, NinS21912, NinS21913, NinS26653, NinS27925 |
| AT1G79340 | MC4 | NinS16882 |
| AT2G39660 | BIK1 | NinS15857 |
| AT3G55450 | PBL1 | None |
| AT1G07570 | PBL9 | None |
| AT2G28930 | PBL10 | NinS00732, NinS15855, NinS20080 NinS23043, NinS26119 |
| AT5G02290 | PBL11 | NinS07993, NinS07998 |
| AT4G01370 | MPK4 | NinS13787, NinS15730 |
| AT1G43700 | VIP1 | NinS05435, NinS05435 |
| AT1G51340 | DTX42 | NinS17054, NinS20376, NinS23574 |
| AT2G21340 | DTX46 | NinS01455 |
| AT1G04610 | YUC3 | NinS21239 |

**
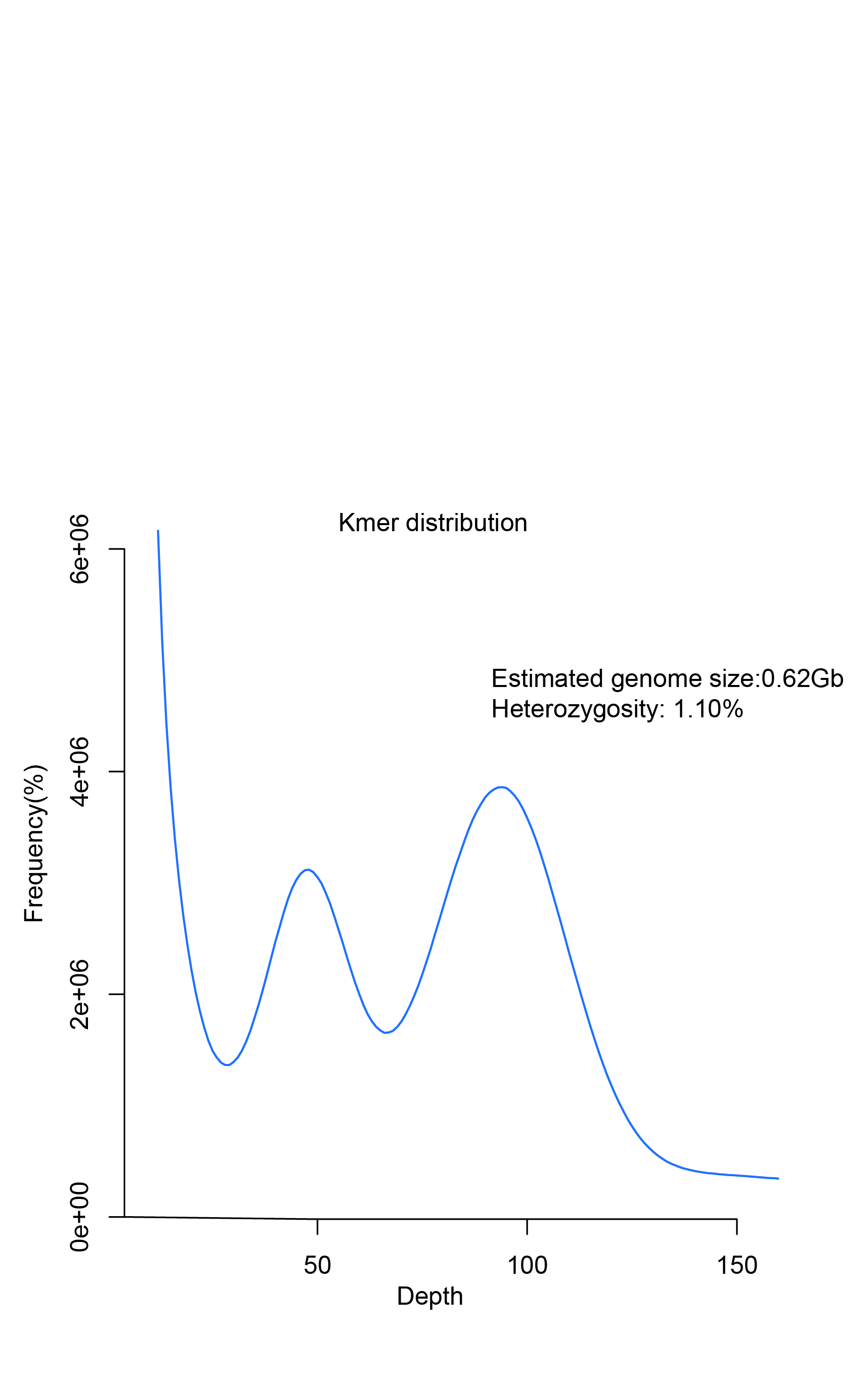
Supplementary Figure 1 *k-mer* (17 *k-mer*) distribution and estimation of genome size and heterozygosity.**

**
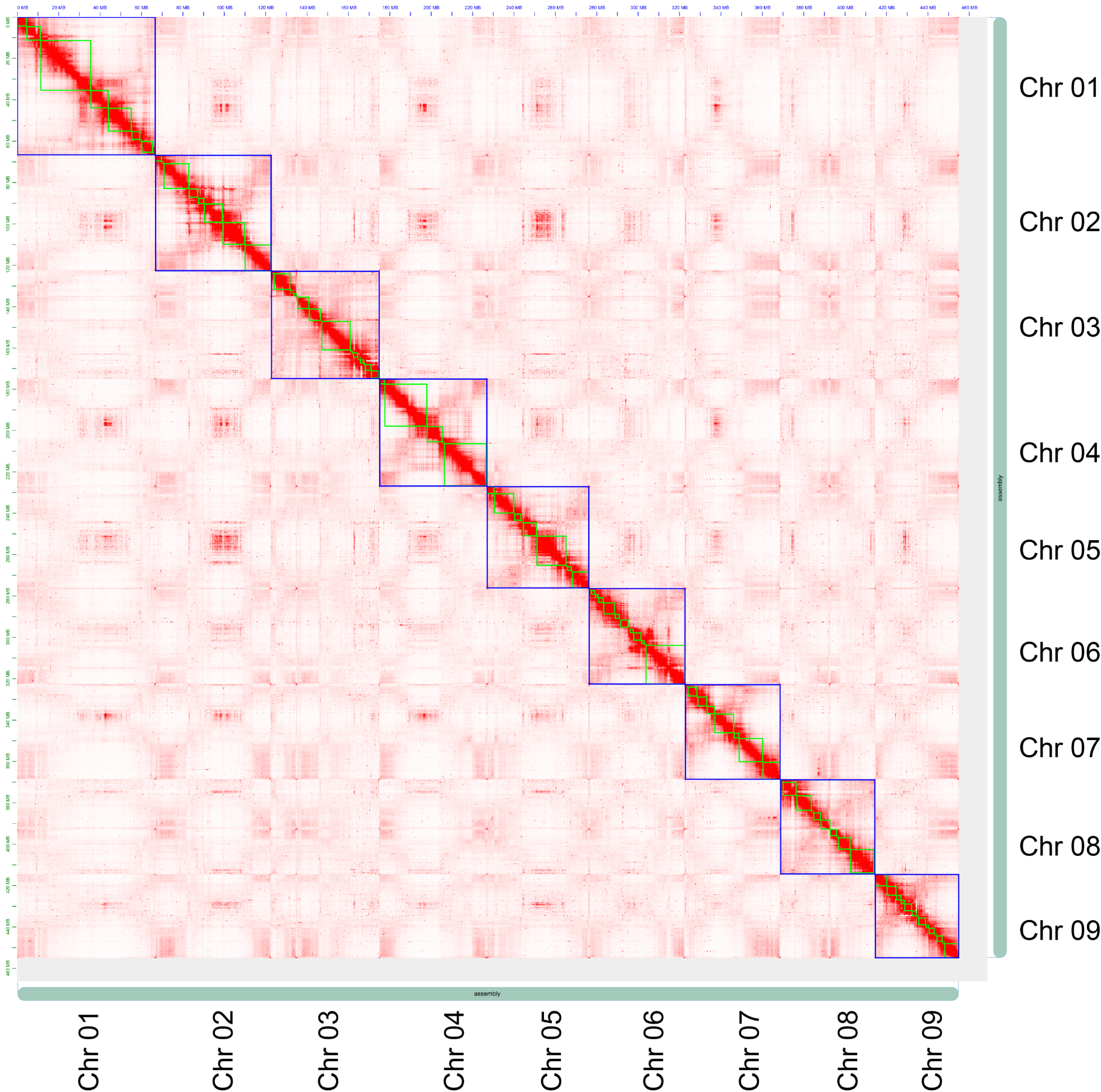
**

**Supplementary Figure 2 Hi-C interactions among nine chromosomes. Strong interactions are indicated in dark red and weak interactions are indicated in light red.**


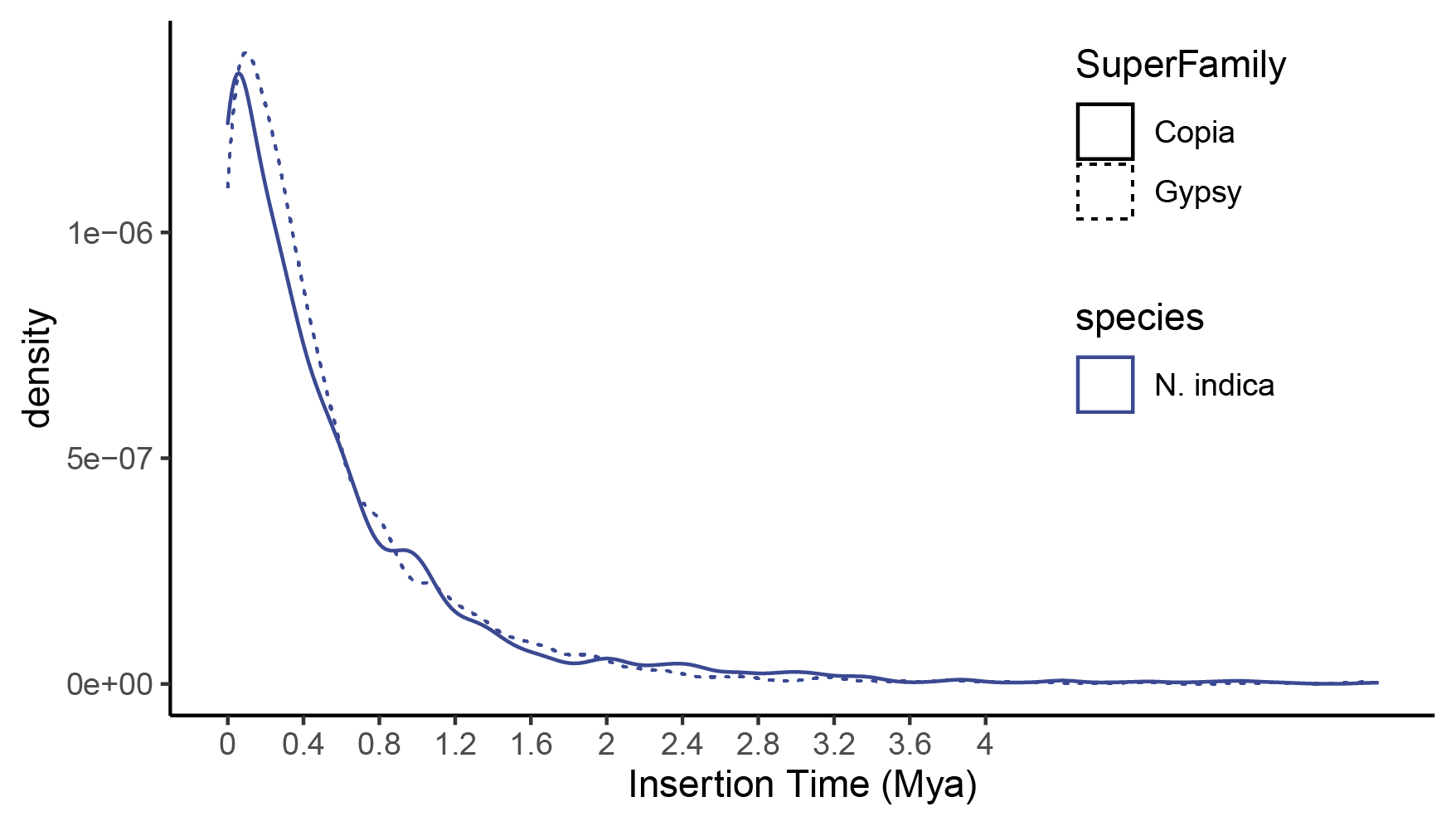


**Supplementary Figure 3 The insertion time distribution of intact LTRs in the *N. indica* genome (Mya indicated million years ago).**


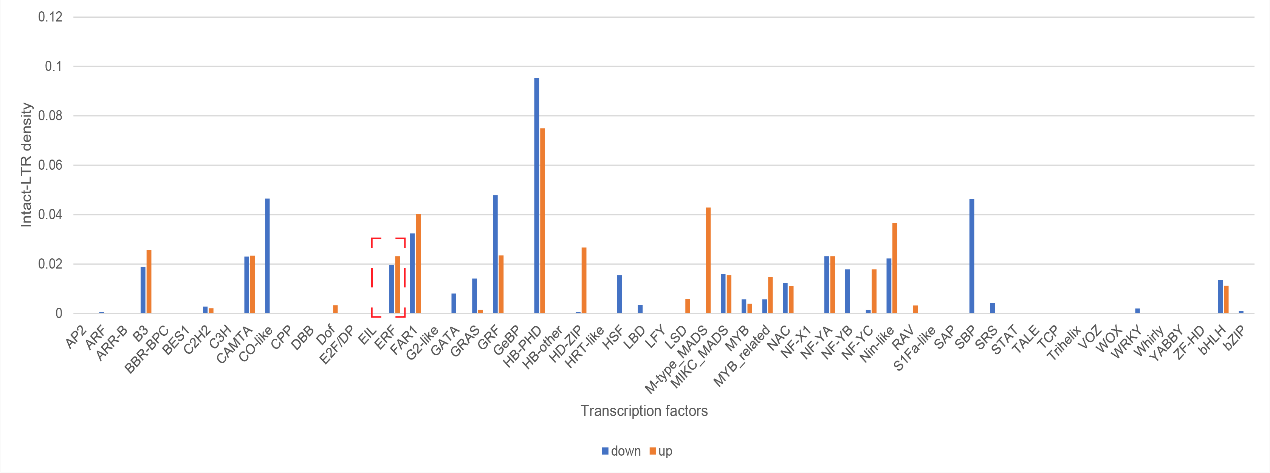


**Supplementary Figure 4 Enrichment of intact-LTRs in the regions 2kb upstream and 2kb downstream of different TF families.**


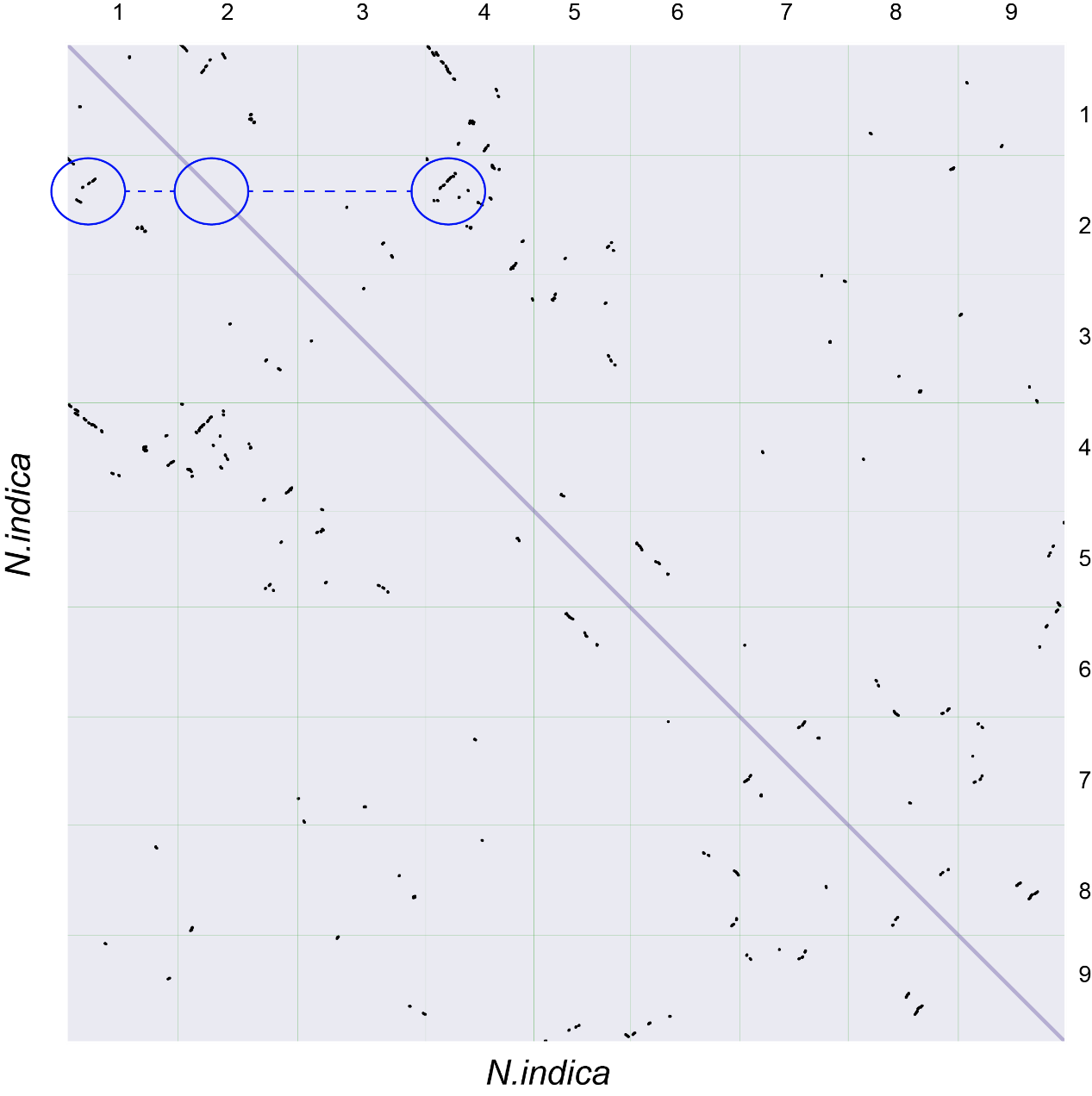


**Supplementary Figure 5 Dotplot analysis of *N. indica* that illustrate WGT-γ event.**


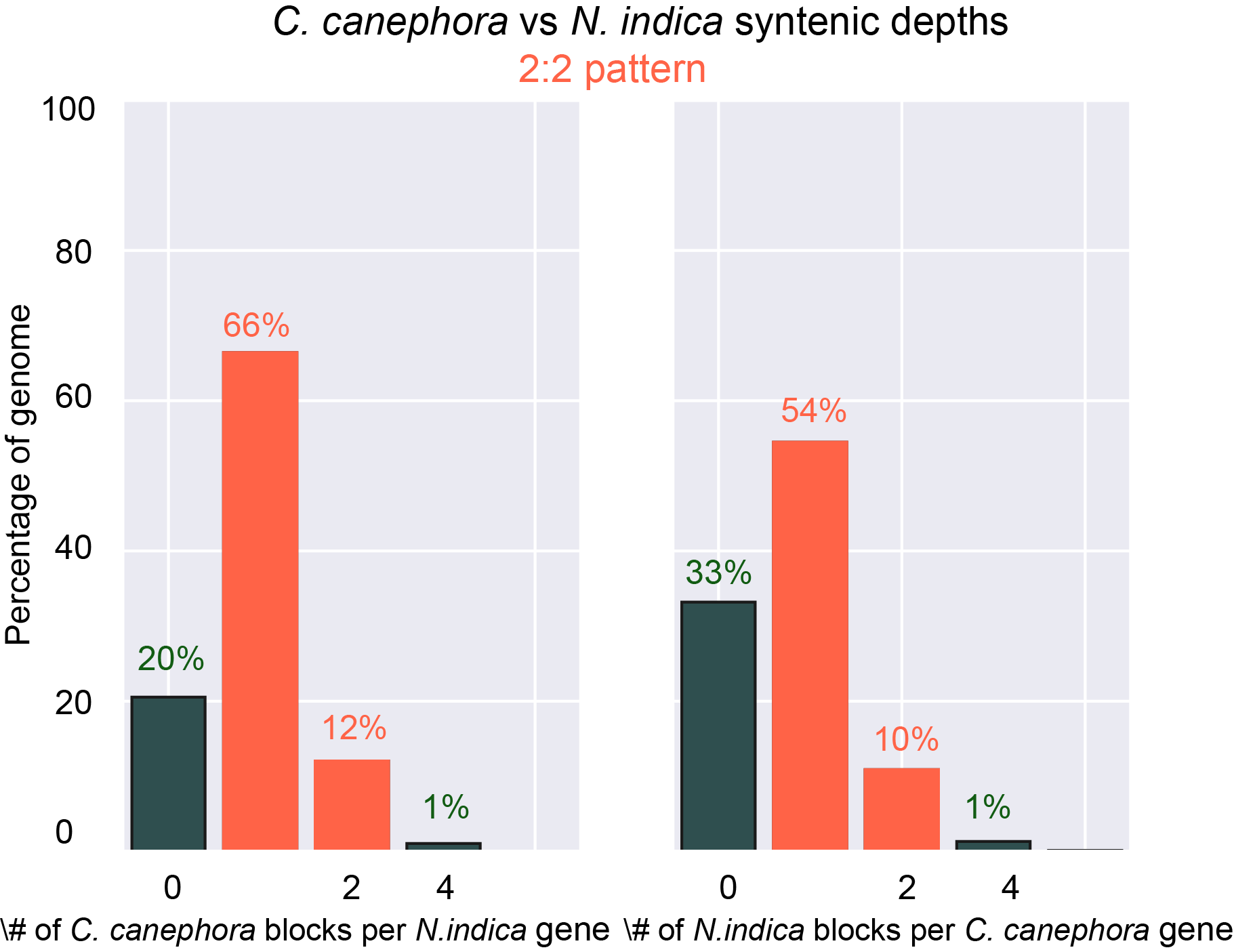


**Supplementary Figure 6 Syntenic depth between coffee and *N. indica* (2:2 syntenic relationship).**


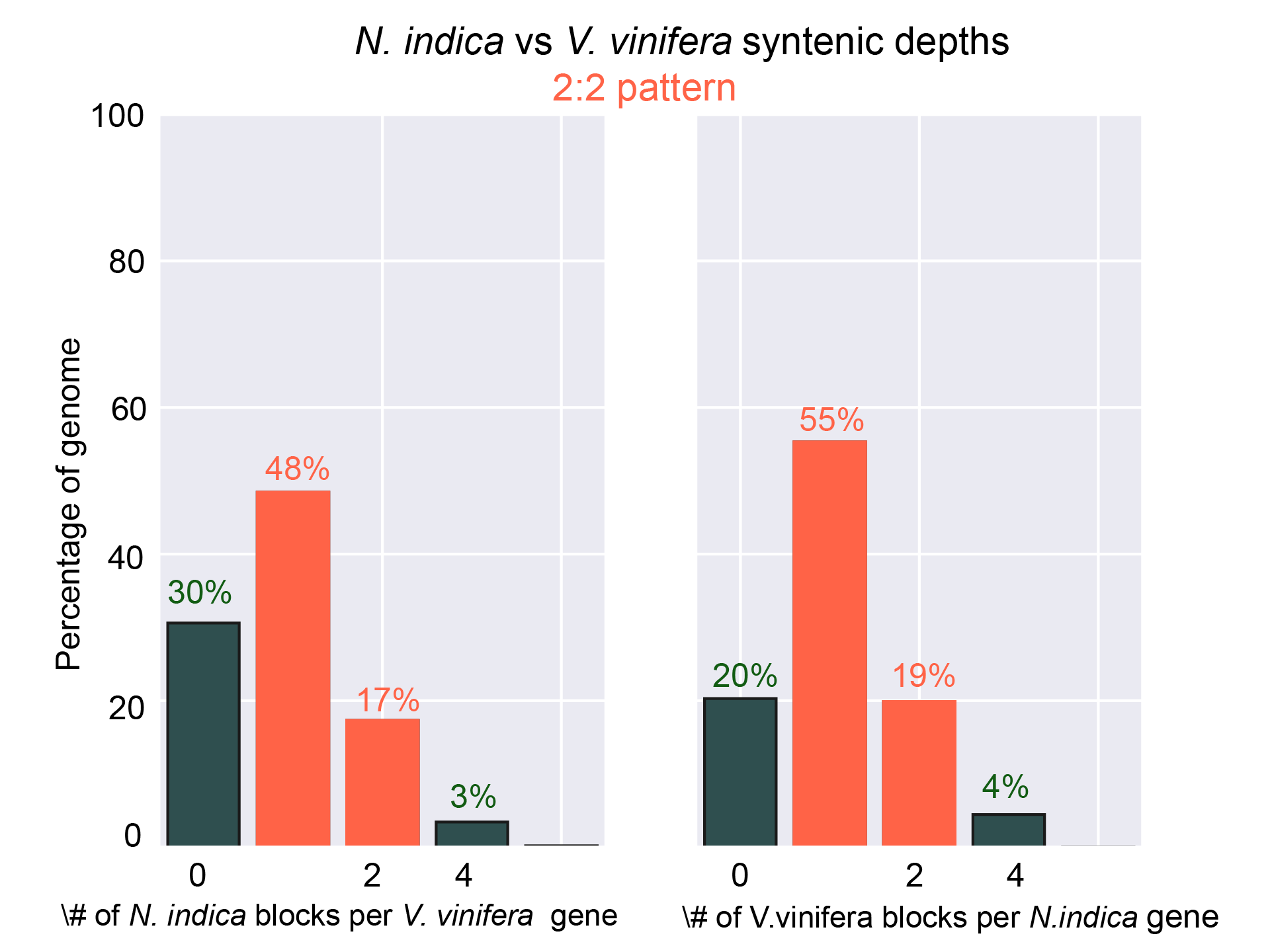


**Supplementary Figure 7 Syntenic depth between *V. vinifera* and *N. indica* (2:2 syntenic relationship).**


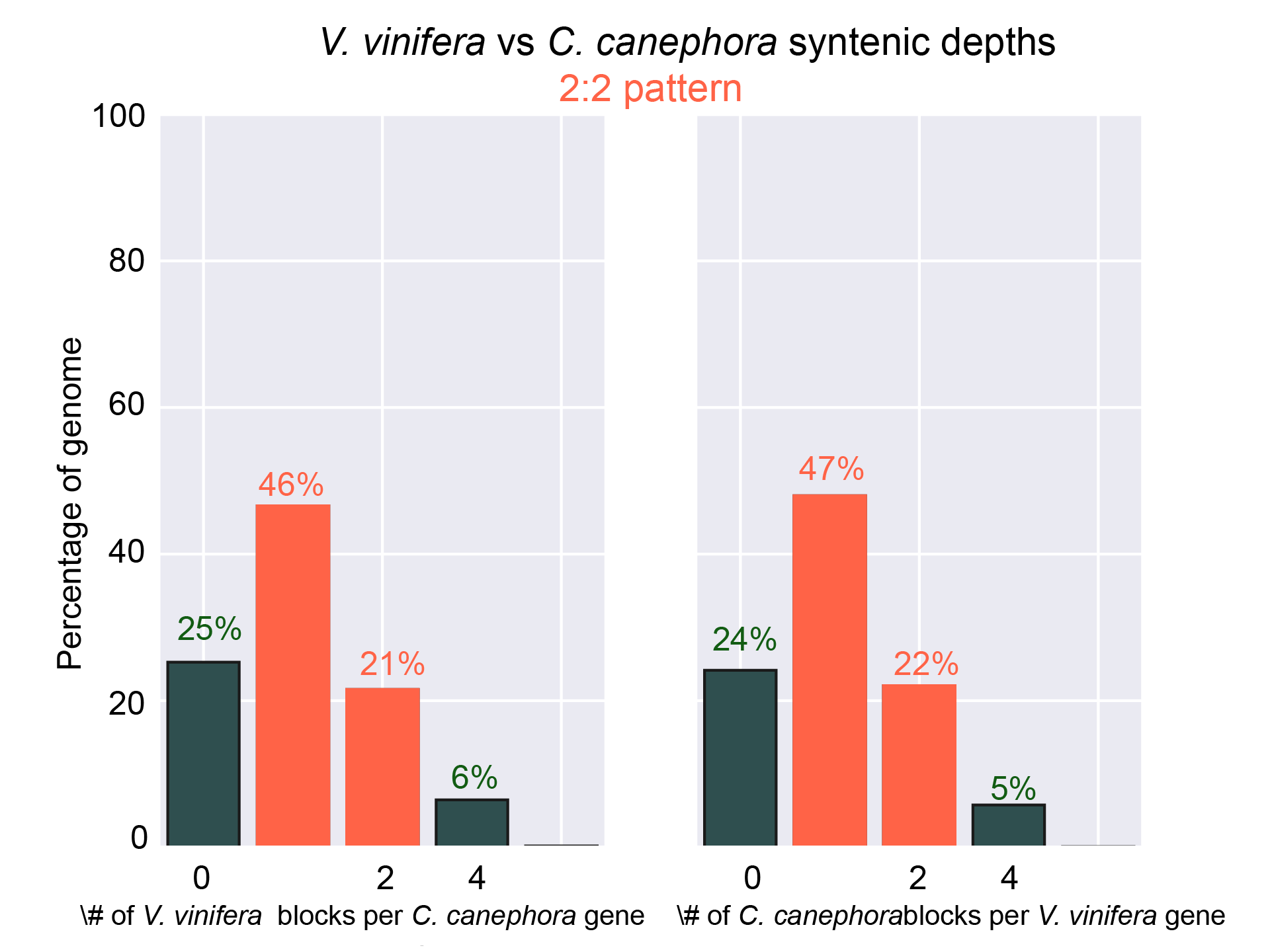


**Supplementary Figure 8 Syntenic depth between *V. vinifera* and *C. cardunculus* (2:2 syntenic relationship).**


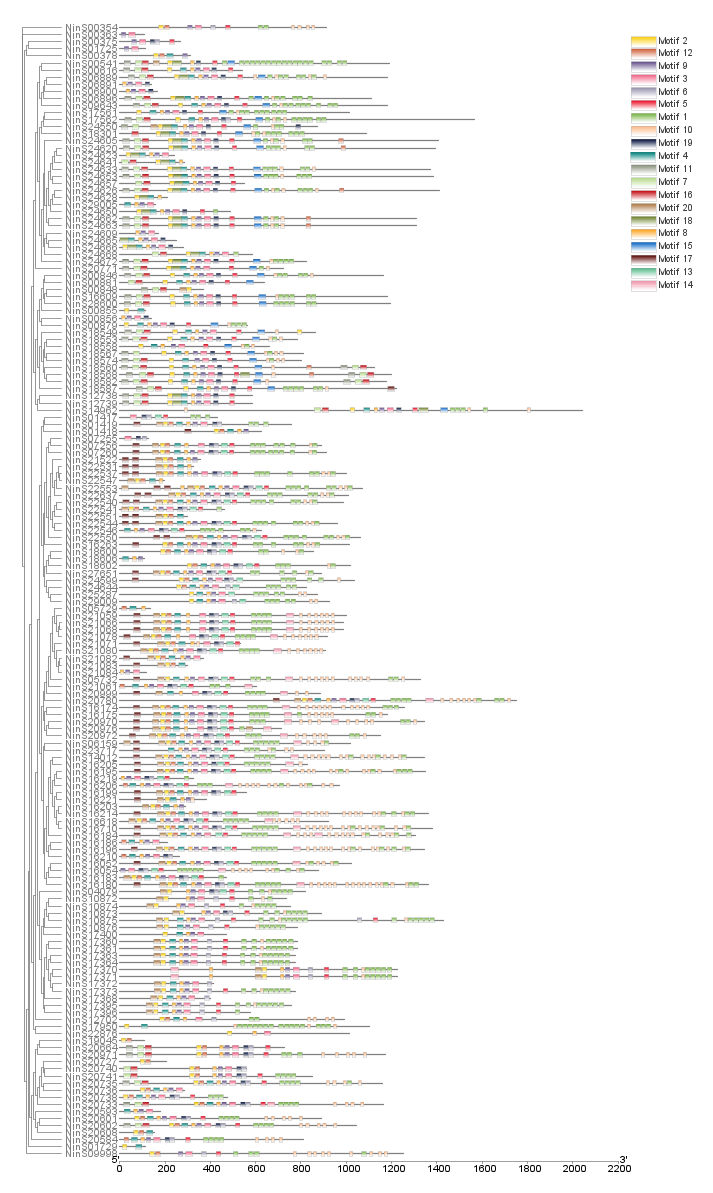


**Supplementary Figure 9 Conserved motif distribution of the *N. indica* NBS-coding proteins.**


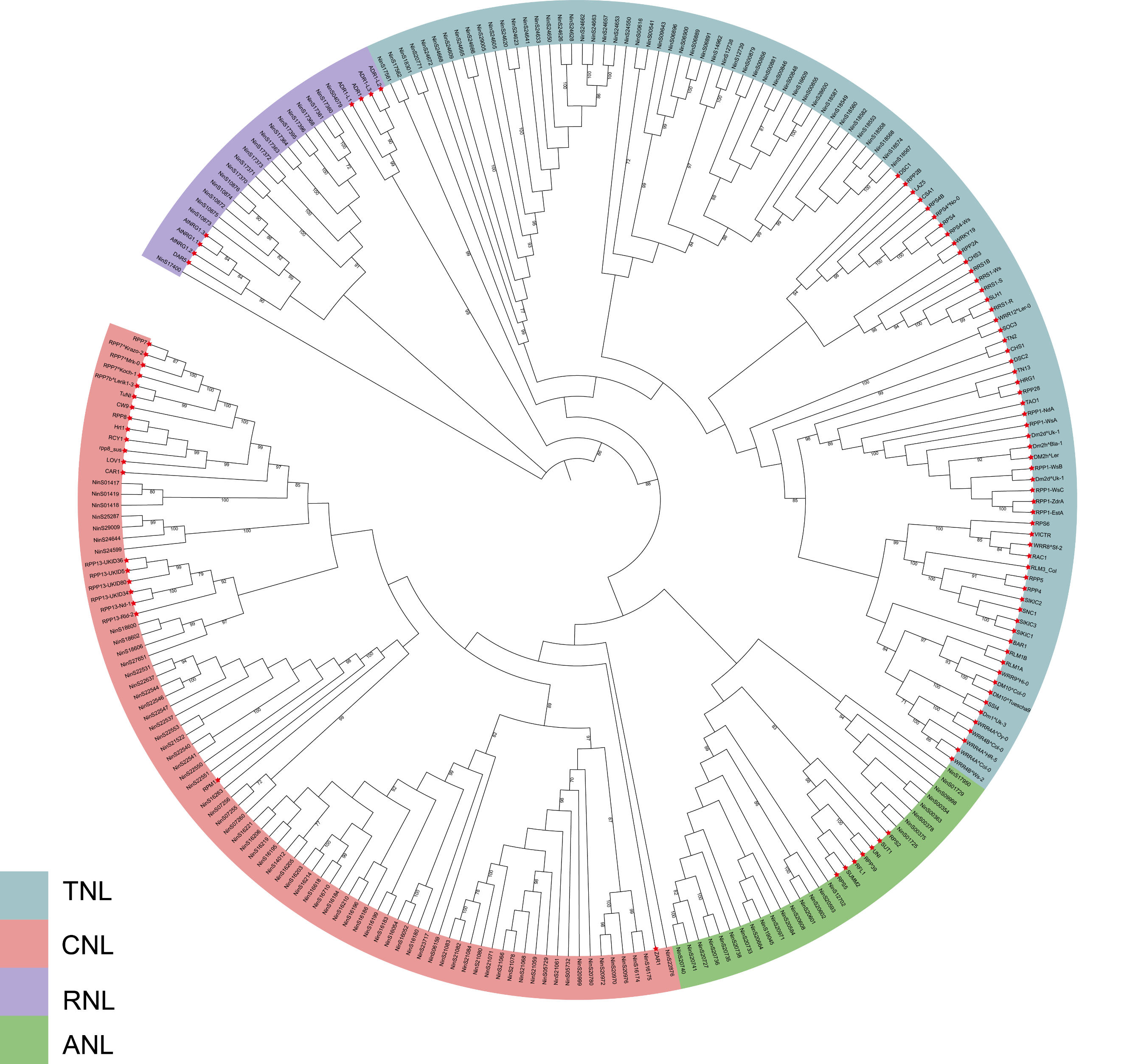


**Supplementary Figure 10 A phylogenetic tree of nucleotide-binding site (NBS) domains in 160 NBS-LRR encoding genes of *N. indica* and 91 functionally validated NBS-LRR encoding genes of *A. thaliana* (Red star represent genes from *A. thaliana*). The tree only shows bootstrap values >70%.**


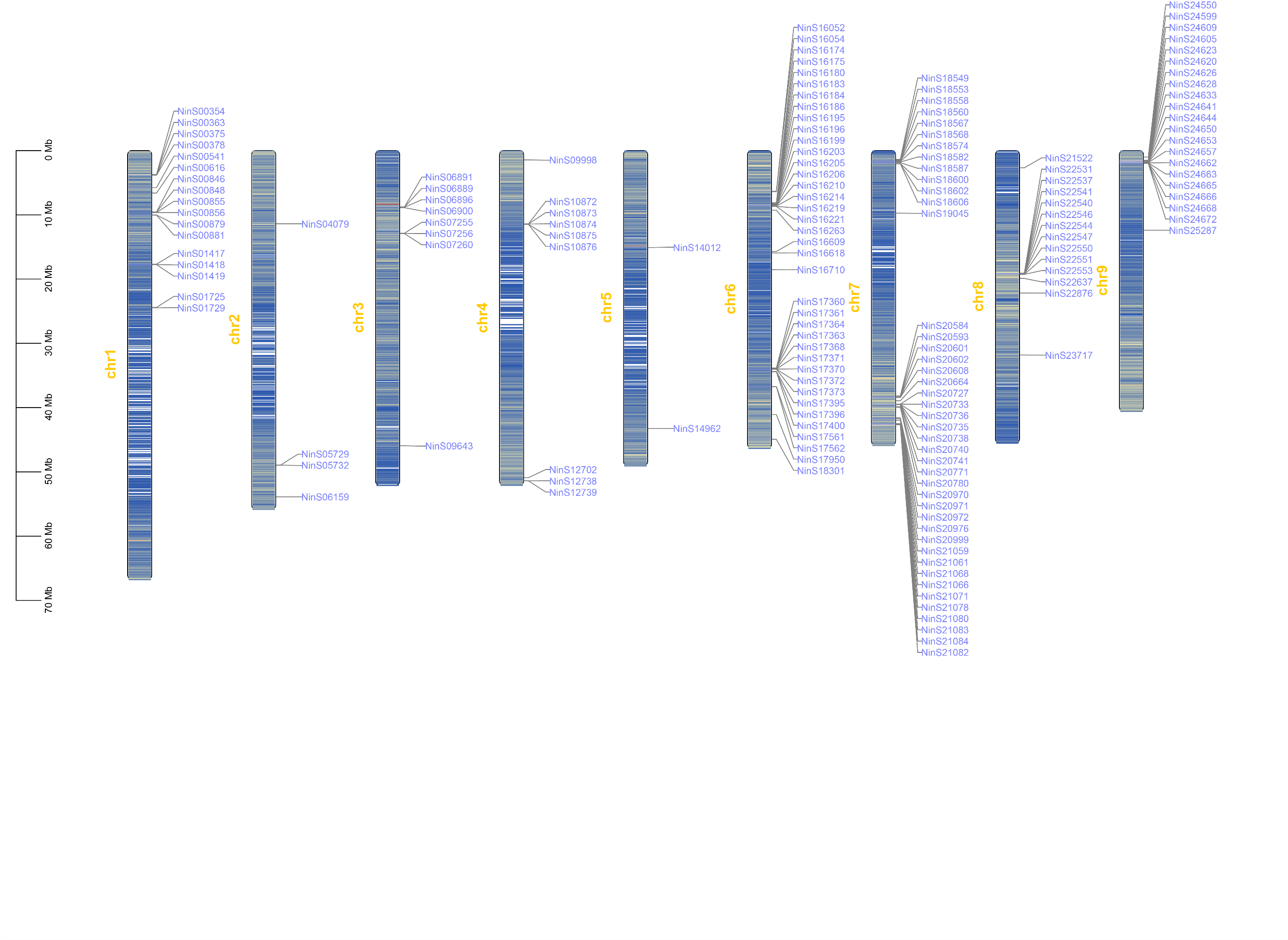


**Supplementary Figure 11 Chromosomal location of NBS-coding genes in the *N. indica* genome.**
